# Progenitor-Derivative Relationships of *Hordeum* Polyploids (Poaceae, Triticeae) Inferred from Sequences of *TOPO6*, a Nuclear Low-Copy Gene Region

**DOI:** 10.1371/journal.pone.0033808

**Published:** 2012-03-30

**Authors:** Jonathan Brassac, Sabine S. Jakob, Frank R. Blattner

**Affiliations:** Taxonomy and Evolutionary Biology, Leibniz Institute of Plant Genetics and Crop Research (IPK), Gatersleben, Germany; University of Gottingen, Germany

## Abstract

Polyploidization is a major mechanism of speciation in plants. Within the barley genus *Hordeum*, approximately half of the taxa are polyploids. While for diploid species a good hypothesis of phylogenetic relationships exists, there is little information available for the polyploids (4×, 6×) of *Hordeum*. Relationships among all 33 diploid and polyploid *Hordeum* species were analyzed with the low-copy nuclear marker region *TOPO6* for 341 *Hordeum* individuals and eight outgroup species. PCR products were either directly sequenced or cloned and on average 12 clones per individual were included in phylogenetic analyses. In most diploid *Hordeum* species *TOPO6* is probably a single-copy locus. Most sequences found in polyploid individuals phylogenetically cluster together with sequences derived from diploid species and thus allow the identification of parental taxa of polyploids. Four groups of sequences occurring only in polyploid taxa are interpreted as footprints of extinct diploid taxa, which contributed to allopolyploid evolution. Our analysis identifies three key species involved in the evolution of the American polyploids of the genus. (*i*) All but one of the American tetraploids have a *TOPO6* copy originating from the Central Asian diploid *H. roshevitzii*, the second copy clustering with different American diploid species. (*ii*) All hexaploid species from the New World have a copy of an extinct close relative of *H. californicum* and (*iii*) possess the *TOPO6* sequence pattern of tetraploid *H. jubatum*, each with an additional copy derived from different American diploids. Tetraploid *H. bulbosum* is an autopolyploid, while the assumed autopolyploid *H. brevisubulatum* (4×, 6×) was identified as allopolyploid throughout most of its distribution area. The use of a proof-reading DNA polymerase in PCR reduced the proportion of chimerical sequences in polyploids in comparison to *Taq* polymerase.

## Introduction

Polyploidization, or whole genome duplication, is a major mechanism in plant evolution. Numerous studies have tried to evaluate the proportion of polyploidy in angiosperms, varying widely between 30 and 80% [1]. It is now acknowledged that probably all angiosperm lineages experienced one or several rounds of polyploidization in their history [2]–[4]. Otto and Whitton [5], analyzing the distribution of haploid chromosome numbers, estimated that polyploidy might be involved in about 2–4% of speciation events, thus proposing that polyploidization “may be the single most common mechanism of sympatric speciation in plants” ([5], p. 427).

Two different concepts for the definition of the type of polyploidy exist. On the one hand is the classic cytogenetic definition where the presence of only bivalent-forming chromosomes during meiosis characterizes allopolyploids while multivalent formation of homoeologous chromosomes indicates autopolyploidy [6], [7]. The second definition is based on a taxonomic concept, where polyploids formed through hybridization of different species (allopolyploids) contrast with hybrids formed through genome duplication or crossing of different genotypes from within a species (autopolyploids). Taxonomic allopolyploids are often termed segmental allopolyploids in the cytogenetic reference frame, indicating the presence of only locally differentiated chromosomes. We here use the taxonomic system of polyploid definition and explicitly refer to cytogenetic allopolyploids by indicating their genome composition.

The grass genus *Hordeum* L. belongs to the economically important tribe Triticeae and consists of 33 species (including cultivated barley, *H. vulgare*) distributed in arid and temperate regions of the world with two centers of diversity in Eurasia and in southern South America [8], [9]. Cytogenetic studies of meiotic chromosome behavior in interspecific hybrids led to the definition of four different genomes within *Hordeum*. For genome denomination we follow Blattner [9], with the **H** genome occurring in *H. vulgare* and *H. bulbosum*, **Xu** in *H. murinum*, **Xa** in *H. marinum* and *H. gussoneanum*, and **I** in the remaining species. The genus is particularly well suited to the study of polyploidization, as nearly half of the species are polyploids, with tetraploid (2*n* = 4*x* = 28) and hexaploid (2*n* = 6*x* = 42) taxa and cytotypes, comprising allo- and autopolyploids [8].

During the last 50 years numerous analyses have been carried out to study relationships in *Hordeum* [9] due to the potential of the wild relatives to improve barley. For the diploid taxa of *Hordeum*, datasets that include multiple nuclear loci converge to similar results [9]. Thus, a good phylogenetic hypothesis seems to be accomplished for this group, although studies including several individuals per species are yet rare. For polyploid species and cytotypes of *Hordeum* phylogenetic relationships were studied mainly for small taxon groups [10]–[13] using nuclear low-copy number loci. Blattner [14] conducted a thorough phylogenetic analysis of all taxa of the genus, including mostly multiple individuals per taxon. This analysis used nuclear rDNA internal transcribed spacer (ITS) sequences as molecular markers, which can undergo unidirectional homogenization [15] or loss of rDNA clusters [16] and thus are not ideal for the study of polyploid evolution. Therefore, a phylogenetic study of all *Hordeum* polyploids based on several individuals per species and a single- or low-copy nuclear locus is still lacking, which severely restricts evolutionary studies in these taxa.

In this study we report results from a phylogenetic analysis of all *Hordeum* species using cloned sequences of the nuclear low-copy region *TOPO6* [12] that is a partial sequence of the *Spo11* gene, a conserved plant homologue of the widespread archaean topoisomerase VI subunit A involved in inducing meiotic DNA double-strand breaks during recombination [17]–[18]. It consists of an unusually high number of introns [19] with exons conserved enough to design PCR primers. To determine genetic diversity of this locus within species and to be able to detect possible independent origins of polyploids we included for all taxa except one (*H. guatemalense*) an average of five individuals per taxon, representing the geographic distribution of the species. The aims of our study are to (*i*) define parental species of allopolyploids, (*ii*) analyze the status of putative autopolyploids, (*iii*) infer single or multiple origins for polyploids, (*iv*) infer speciation events on the polyploid level, (*v*) check for indications of introgression from outside of *Hordeum*, and (*vi*) compare the influence of two different types of DNA polymerases in PCR on the results of polyploid analyses. We do not assume that the gene tree obtained from the analyzed locus will represent the ‘true’ species phylogeny of the genus. This will, however, not impair the objectives of our study, as we are mainly interested in progenitor–derivative relationships between diploids and polyploids, which should be independent from the gross topology of the diploids in a phylogenetic tree.

## Materials and Methods

### Plant materials

We included 341 individuals representing all 33 species and most subspecies of the genus plus one individual of each of eight diploid Triticeae species outside *Hordeum* as outgroups (Table 1). Included individuals were obtained from germplasm repositories or sampled from natural populations (Table S1) and all necessary permits were acquired to use these materials. Herbarium vouchers of the analyzed materials were deposited in the herbaria of the IPK Gatersleben (GAT) or the Museum of Natural History, Buenos Aires (BA).

**Table 1:** Taxa included in the study.

Taxon	Ploidy level (N)^1^	Haploid genome	Native distribution area
***Hordeum*** ** subgenus ** ***Hordeum***			
**Section ** ***Hordeum***			
*H. vulgare* L.^2^			
subsp. *spontaneum* (C.Koch.) Thell.	2× (2)	**H**	SW Asia
*H. bulbosum* L.	2× (5), 4× (3)	**H, HH**	Mediterranean to C Asia
**Section ** ***Trichostachys*** ** Dum.**			
*H. murinum* L.			
subsp. *glaucum* (Steud.) Tzvel.	2× (3)	**Xu**	Mediterranean to C Asia
subsp. *murinum*	4× (3)	**XuXu**	NW Europe to Caucasus
subsp. *leporinum* (Link) Arc.	4× (4), 6× (3)	**XuXu, XuXuXu**	Mediterranean to C Asia
***Hordeum*** ** subgenus ** ***Hordeastrum*** ** (Doell) Rouy**			
**Section ** ***Marina*** ** (Nevski) Jaaska**			
*H. gussoneanum* Parl.	2× (4), 4× (6)	**Xa, XaXa**	Mediterranean to C Asia
*H. marinum* Huds.	2× (3)	**Xa**	Mediterranean
**Section ** ***Stenostachys*** ** Nevski**			
**Series ** ***Sibirica*** ** Nevski**			
*H. bogdanii* Will.	2× (41)	**I**	C Asia
*H. brevisubulatum* (Trin.) Link^2^	2× (5), 4× (9), 6× (4)	**I, II, III**	C Asia
*H. roshevitzii* Bowden	2× (17)	**I**	C Asia
**Series ** ***Critesion*** ** (Raf.) Blattner**			
*H. californicum* Covas & Stebb.	2× (13)	**I**	SW California
*H. chilense* Roem. & Schult.	2× (22)	**I**	Chile and W Argentina
*H. comosum* Presl	2× (24)	**I**	S Argentina
*H. cordobense* Bothmer et al.	2× (22)	**I**	C Argentina
*H. erectifolium* Bothmer et al.	2× (1)	**I**	C Argentina
*H. euclaston* Steud.	2× (14)	**I**	C Argentina, Uruguay
*H. flexuosum* Steud.	2× (8)	**I**	E+C Argentina
*H. intercendens* Nevski	2× (7)	**I**	SW California, NW Mexico
*H. muticum* Presl	2× (10)	**I**	C to N Andes
*H. patagonicum* (Haum.) Covas^2^	2× (14)	**I**	S Argentina
*H. pubiflorum* Hook.f.^2^	2× (17)	**I**	S Argentina
*H. pusillum* Nutt.	2× (13)	**I**	C+E USA
*H. stenostachys* Godr.	2× (19)	**I**	C Argentina
*H. depressum* (Scribn. & Sm.) Rydb.	4× (4)	**II**	W USA
Interserial allopolyploids of series *Critesion* and *Sibirica*			
*H. brachyantherum* Nevski	4× (3)	**II**	W North America, Kamchatka, Newfoundland
*H. fuegianum* Bothmer et al.	4× (3)	**II**	S Argentina, S Chile
*H. guatemalense* Bothmer et al.	4× (1)	**II**	Guatemala, S Mexico
*H. jubatum* L.	4× (4)	**II**	NE Asia, NW+W North America, C Argentina
*H. tetraploidum* Covas	4× (4)	**II**	C Argentina
*H. arizonicum* Covas	6× (3)	**III**	SW USA
*H. lechleri* (Steud.) Schenk	6× (7)	**III**	C+S Argentina
*H. parodii* Covas	6× (4)	**III**	C Argentina
*H. procerum* Nevski	6× (4)	**III**	S Argentina
**Section ** ***Nodosa*** ** (Nevski) Blattner**			
*H. brachyantherum* Nevski	6× (2)	**IIXa**	C California
*H. capense* Thunb.	4× (2)	**IXa**	S Africa
*H. secalinum* Schreb.	4× (4)	**IXa**	Mediterranean to W Europe
**Outgroup species**			
*Dasypyrum villosum* (L) Candargy	2× (1)	**V**	
*Eremopyrum triticeum* (Gaertn.) Nevski	2× (1)	**FXe**	
*Psathyrostachys juncea* (Fisch.) Nevski	2× (1)	**Ns**	
*Taeniatherum caput-medusae* (L.) Nevski	2× (1)	**Ta**	
*Triticum monococcum* L.	2× (1)	**A^m^**	
*Triticum urartu* Tumanian ex Gandilyan	2× (1)	**A^u^**	
*Secale strictum* (C. Presl) C. Presl	2× (1)	**R**	
*Secale vavilovii* Grossh.	2× (1)	**R**	

1 Number of individuals included per species or cytotype;

2 species with subspecies not further detailed here.

### Molecular methods

Genomic DNA was extracted from approximately 10 mg of silica gel-dried leaves with the DNeasy Plant Mini Kit (Qiagen) according to the protocol of the manufacturer. DNA quality and concentrations were checked on 1% agarose gels.

*TOPO6* was amplified as described in Jakob and Blattner [12] using primers Top6-15F (5′-GTG YTG TST YCA ACT GAA GTC-3′) and Top6-17R (5′-CGT ACT CCA RYG CCA TTT C-3′) designed to bind in exons 15 and 17 of the gene. Thus the amplification products consist of introns 15 and 16 together with exon 16, and are of lengths between 800 and 1200 base pairs (bp) in many pooid grasses (Blattner, unpublished). PCR was conducted for all but 12 of the analyzed individuals, using 1 U of a standard DNA polymerase (Qiagen Taq DNA polymerase) in 50 µl reaction volume containing approximately 10–50 ng of genomic DNA, 1× Coral Load PCR Buffer (Qiagen), 1× Q Solution (Qiagen), 1.25 mM MgCl_2_, 0.2 mM of each dNTP, and 0.5 µM of each primer. The amplification process consisted of initial denaturation for 2 min at 95°C, followed by 35 cycles of 30 sec at 96°C, 1 min at 56°C, 2.3 min at 72°C, and a final extension of 12 min at 72°C. To reduce PCR errors, which become visible when amplicons are cloned, amplification of *TOPO6* was performed in 27 polyploids and five recalcitrant diploid individuals using 1 U proof-reading polymerase (Finnzymes OY, Phusion Hot Start DNA polymerase) with the same PCR conditions as before but using the supplied 1× Phusion HF Buffer. Amplification conditions were modified as suggested by the provider with higher denaturation (98°C) and annealing temperatures (59°C).

Amplicons were purified using Nucleofast 96 Spin Plates (Macherey-Nagel) according to the protocol of the manufacturer, eluted in 20 µl of TE buffer, and sequenced on an ABI 3730XL automatic DNA sequencer (Applied Biosystems). For most of the diploid *Hordeum* and outgroup species amplicons were directly sequenced, while for all *Hordeum* polyploids and eight diploids amplicons were ligated into the pJET1.2 vector (Fermentas) and transformed into DH5α *E. coli* strains. On average 15 colonies per individual were randomly selected for screening the insertion of a *TOPO6* fragment via PCR employing the primers pJET-F and pJET-R (Fermentas). Colonies showing products of the correct size (about 900 bp) were transferred to 200 µl LB broth medium with 0.1 mg/ml ampicilline and incubated overnight at 37°C. For a total of 952 colonies plasmids were isolated, and for each clone 1 µl was used for sequencing forward and reverse strands as described in Blattner [14] using primers pJET-F and pJET-R. For eight diploid individuals six to ten clones were sequenced to test if *TOPO6* is a single-copy locus, while for all polyploids an average of 12 clones each were sequenced.

### Data analyses

Manual editing of sequences and multiple sequence alignments were performed with Geneious Pro v5.4 [20] followed by manual adjustments of the alignments. Most sequences obtained by cloning of amplicons of regular *Taq* polymerase from single individuals had very similar sequences, differing in one to ten mutations (on average two), which were not shared by more than one clone. These differences are very likely PCR errors from the *Taq* polymerase that occur during cloning or sequencing. In these cases consensus sequences for highly similar sequences were created in order to reduce the number of singletons in the alignment.

Chimerical sequences can be the result of natural recombination between alleles of orthologous or homoeologous genes and/or PCR-mediated recombination. PCR recombination occurs when partially extended PCR fragments function as a primer to amplify divergent sequences [21]. Allopolyploid species are especially prone to the formation of chimerical sequences due to the presence of two or more homoeologous copies. Bifurcating phylogenetic trees cannot represent precisely the evolutionary histories of recombinant sequences and the presence of chimerical sequences disturbs analysis algorithms, as they combine signals from different phylogenetic groups. Automated methods included in Rdp3 [22] to account for recombination events were used but the results were not conclusive due to the high number of PCR-mediated mutations present in the raw data set. Therefore, sequences were thoroughly inspected by eye to identify sequences showing combinations of polymorphic sites present in different alleles (Fig. S1). The recombinant sequences were excluded from the data set prior to the analysis. In cases of identical sequences derived from the same individual only one sequence was included in the data analysis.

After a preliminary analysis, out of the 341 individuals sequenced, a reduced dataset representative of all the diversity found was used for the different analyses. Thus alleles shared by more than one individual per species were included only once. The final alignment consisted of 278 sequences. This dataset (File S1) and a subset containing only the sequences derived from diploid species and cytotypes consisting of 109 sequences (File S2) were analyzed using parsimony and Bayesian methods. In all analyses *Psathyrostachys juncea* was defined as outgroup [23]. A maximum parsimony (MP) analysis was conducted in Paup* 4b10 [24] using the two-step procedure described in Blattner [14]. In an initial heuristic search with 1000 random addition sequences and TBR branch swapping the number of trees retained was restricted to five per random addition. The best trees from this search were used as starting trees in a second heuristic search utilizing TBR branch swapping, restricting the number of saved trees to 50,000. To test the statistical support of clades a bootstrap analysis with 50,000 re-samples and the fast-and-stepwise algorithm was conducted in Paup*.

For Bayesian inference (BI), different models of sequence evolution were investigated with MrModeltest version 2.3 [25]. As partitioning of the data to account for intron/exon differences did not change the outcome of an initial BI analysis we inferred an overall model of sequences evolution for the entire marker region. Among the 24 models tested, the best-fit model selected by the hierarchical Likelihood Ratio Test (hLRT; [26]) and Akaike Information Criterion (AIC; [27], [28]) was HKY+Γ, a transition/transversion model [29] with rates variation according to a gamma distribution [30]. The analysis conducted with MrBayes version 3.1.2 [31] consisted of two parallel Metropolis coupled Monte Carlo Markov chain analyses with six chains per run for 5 million generations and sampling trees every 1000 generations. The temperature parameter was set at 0.05 to obtain a value of state swap frequency within the range of 10% to 70%. The convergence of the parameters was evaluated with the standard deviation of split frequencies (<0.01) and with the program Tracer version 1.5 [32]. The topology convergence was checked using the compare function of the online application Awty [33], which plots posterior probabilities of clade support values for both runs against each other. The first 25% trees were discarded as burn-in and a consensus tree was computed in MrBayes 3.1.2.

To visualize species relationships, the final analysis was summarized in a schematic tree as used by Blattner [14]. In this scheme, the phylogeny of sequences derived from diploid species was used as a backbone, and the polyploid species were connected to the diploids according to the placement of their sequences in the complete analysis.

## Results

**Figure 1:**
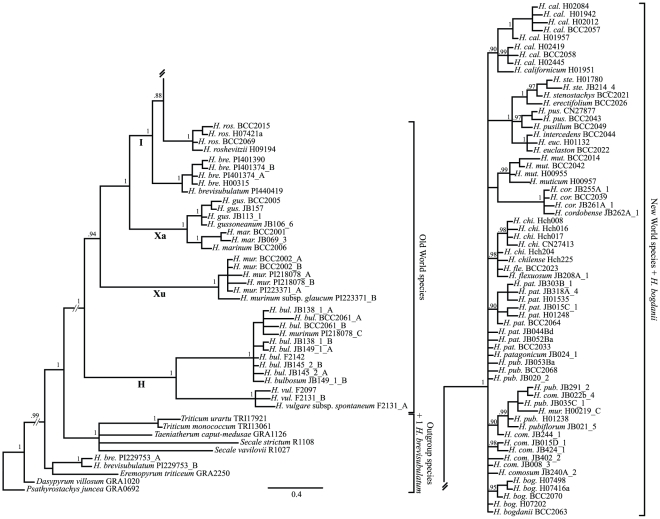
Phylogenetic tree derived from *TOPO6* sequences of the diploid *Hordeum* taxa and eight outgroup species calculated with Bayesian inference. Posterior probability values of the clades are indicated along the branches. Bold letters depict genome denominations following Blattner (2009). After the species name and individual number the different copies found per individual are indicated (A–C) in case of cloned sequences.

**Figure 2:**
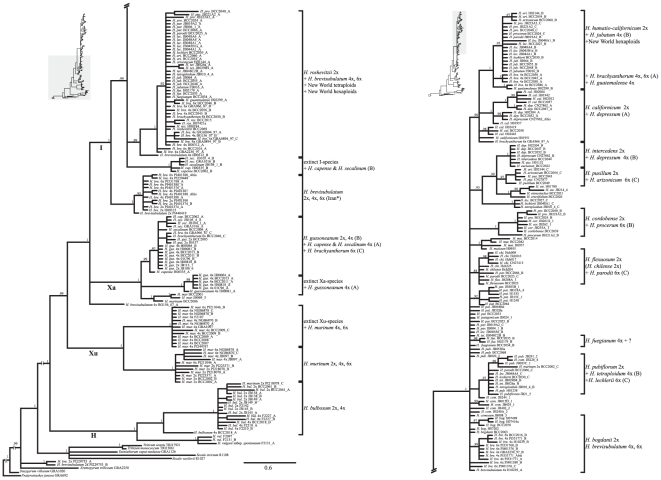
Phylogenetic tree derived from cloned *TOPO6* sequences from diploid and polyploid *Hordeum* taxa and eight outgroup species calculated with Bayesian inference. Posterior probability values of the clades are indicated along the branches. Clades containing diploid and polyploid-derived sequences are indicated to the right. Genome denominations are given in bold type.

**Figure 3:**
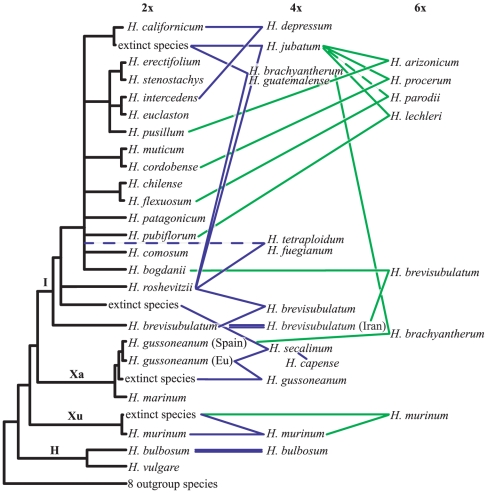
Scheme summarizing phylogenetic relationships of species and cytotypes in the genus *Hordeum* based on *TOPO6*. Diploid taxa were drawn directly at the tree, while tetra- and hexaploids were connected by lines to their inferred parental taxa. Dashed lines indicate uncertainties and double lines depict an autopolyploid origin. Colors refer to the ploidy level of the taxa (tetraploid blue lines, hexaploid green lines).

The *TOPO6* sequences obtained in this study varied in lengths between 868 and 1057 bp and were stored in the EMBL nucleotide database under accession numbers HE655746-HE656023. The alignment of 278 *TOPO6* sequences was 1275 bp long and contained 367 variable sites (281 for diploids only), of which 248 were parsimony-informative (205 for diploids only). All analysis algorithms resulted in very similar tree topologies, thus only the BI trees are presented (Fig. 1 for diploids only, Fig. 2 for the complete dataset), while results of the MP analyses are available as Figures S2 and S3. Both analyses are summarized in a scheme of the *TOPO6*-based species and cytotype relationships within *Hordeum* (Fig. 3). All analyses revealed the sequences derived from *Hordeum* species to be monophyletic with one exception (Fig. 1): two clone sequences from a single diploid *H. brevisubulatum* individual (PI229753) clustered outside the *Hordeum* clade, together with *Eremopyrum triticeum*. Sequences from the four genome groups in *Hordeum* (**H**, **I**, **Xa**, **Xu**) were mainly found monophyletic (Fig. 1) with few exceptions: (*i*) the *H. brevisubulatum* sequences already mentioned, (*ii*) a tetraploid individual of *H. brevisubulatum* (BG156/07) having one sequence falling outside of the **I** clade in a polytomy together with the **Xa**+**I** clade, and (*iii*) two diploid individuals of *H. murinum* (**Xu** genome, [12]) with two clones clustering with **H**-genome *H. bulbosum* (derived from *H. murinum* PI218078) and one clone clustering with **I**-genome *H. pubiflorum* (from *H. murinum* BCC2002). The *Hordeum* clade received strong support with a posterior probability (pp) of 0.99 in BI. The **H**-genome sequences formed the sistergroup to the remaining species, with **Xu**-genome sequences grouping as sister to the clade including sequences derived from **Xa**- and **I**-genome taxa.

In the **H**-genome clade (section *Hordeum*), sequences of both cytotypes (2× and 4×) of *H. bulbosum* clustered together (Fig. 2) in one strongly supported clade (1.0 pp). Between one and three similar sequences were recovered per individual (Figs. 1 and 2), indicating either duplication of the *TOPO6* locus in this species or strong allelic diversity within individuals. We did, however, retrieve no sequences indicating that a taxon outside the extant diploid of *H. bulbosum* contributed to the formation of the tetraploid, which confirms the autopolyploid origin of this cytotype.

The **Xu**-genome group (section *Trichostachys*) of the *H. murinum* taxon complex consisted of one clade (1.0 pp) in the analysis of diploids (*H. murinum* subsp. *glaucum*) and two clades when polyploids were included (Figs. 1 and 2). This second clade was formed only by sequences derived from polyploids, indicating the existence of homoeologues not occurring in extant diploid individuals [12].

In the **Xa**-genome clade (section *Marina*), sequences clustered in three strongly supported groups (Fig. 2): (*i*) *H. marinum* sequences (1.0 pp), (*ii*) *H. gussoneanum* (2× and 4×, type B), *H. capense* (type A), *H. secalinum* (type A) and hexaploid *H. brachyantherum* (type C) with 0.98 pp, and (*iii*) *H. gussoneanum* sequences derived from tetraploids only (type A, 1.0 pp). As before, this latter group indicates homoeologues that do not occur in extant diploid individuals of section *Marina*.

The **I**-genome group (section *Stenostachys*) consisted of the Asian species *H. brevisubulatum* and *H. roshevitzii* being successive sister taxa to the large clade of sequences from Asian *H. bogdanii* together with all sequences derived from American species (Fig. 1). In this latter clade sequences of single species and species groups were arranged along a large polytomy. *Hordeum bogdanii* and *H. californicum* sequences as well as sequences of the three closely related Patagonian species *H. comosum*, *H. patagonicum*, and *H. pubiflorum* occured directly along this polytomy or formed clades of sequences derived from single species, while sequences from the other species formed mixed clades. Thus, *H. chilense* grouped together with *H. flexuosum*, *H. cordobense* with *H. muticum*, and *H. euclaston* with *H. intercedens*, *H. pusillum*, *H. erectifolium*, and *H. stenostachys* (Fig. 1). The sequences derived from *H. bogdanii* possessed a 33 bp region (alignment positions 925–957) shared by all Old World *Hordeum* species and absent in the American species (Fig. S4).

In the tree including polyploid-derived sequences (Fig. 2) in the **I**-genome group a clade (1.0 pp) consisting only of sequences from the three cytotypes (2×, 4× and 6×) of *H. brevisubulatum* was sister to a large clade with a basal trichotomy. Its first clade (1.0 pp) contained only sequences of *H. capense* and *H. secalinum* (type B) without any sequences from an extant diploid species. The second clade (0.99 pp) consisted of sequences of tetraploid and hexaploid *H. brevisubulatum* together with sequences originating from the diploid species *H. roshevitzii*, the tetraploid species *H. jubatum*, *H. brachyantherum*, *H. guatemalense*, *H. tetraploidum*, and *H. fuegianum*, and the hexaploid species *H. arizonicum*, *H. brachyantherum*, *H. lechleri*, *H. parodii*, and *H. procerum*. In the third clade, in addition to the diploid-derived sequences, Asian polyploid *H. brevisubulatum* and all American polyploids grouped mostly together with specific diploids into smaller subclades. Thus, *H. tetraploidum* (4×), *H. lechleri* (6×) and *H. parodii* (6×) fell in a clade with diploid *H. pubiflorum* and, augmented by *H. fuegianum* (4×), also in the group of sequences along the basal polytomy. *Hordeum parodii* (6×) grouped together with diploid *H. flexuosum*, *H. procerum* (6×) with diploid *H. cordobense*, *H. arizonicum* (6×) with diploid *H. pusillum*, *H. depressum* (4×) with diploid *H. intercedens*, and *H. depressum* (4×) and *H. brachyantherum* (6×) also with diploid *H. californicum*. Finally, there is a clade consisting of sequences found in *H. brachyantherum* (4×), *H. guatemalense* (4×), *H. jubatum* (4×) and all American hexaploid species. In this latter clade no sequences derived from any extant diploid species could be found.

For some tetraploid Iranian individuals of *H. brevisubulatum* we got no indication that other taxa than diploid *H. brevisubulatum* contributed *TOPO6* sequences, which indicates an autopolyploid origin of these individuals.

The *TOPO6* alignment revealed the presence of two insertions of transposable elements (TE). Thus, a TE of variable size ranging from 78 bp (in *Secale*) to 123 bp (in *Triticum*) is present at alignment positions 968–1092 of the outgroup species. BLAST searches of this element retrieved sequences featuring a *Stowaway* miniature inverted repeat transposable element (MITE). All *Hordeum* species plus *Dasypyrum villosum* and *Eremopyrum triticeum* were missing this element. In addition, the *H. bulbosum* accession BCC2018 had an allele with an insertion of 162 bp similar to a *Stowaway* MITE annotated as *Thalos* (EMBL sequence accession number AF521177.1, position 76813–76977; 92% similarity) that is located in a TA target site (alignment positions 709–872).

### Recombinant clones of *TOPO6* and comparison of two polymerases

Among the 945 clone-derived sequences obtained for this study, 365 from 19 tetra- or hexaploid individuals could be analyzed for the influence of proof-reading versus standard DNA polymerases. Separate PCRs were conducted for these individuals, with both standard and proof-reading enzymes. An example of the resulting sequences is shown in Figure S1. For this subset, *Taq* resulted in an average of 40% (SD = 0.24) of chimerical sequences, while the Phusion Hot Start DNA Polymerase resulted in only half of this proportion (22%, SD = 0.19). This difference was significant (paired Student's t-Test, p = 0.01) when testing for a higher proportion of chimerical sequences with the regular *Taq* than with the Phusion enzyme. Concerning only the tetraploids (10 individuals), we found a significant difference (paired Wilcoxon signed rank test, p = 0.04) with the same alternative hypotheses. In the case of hexaploids (nine individuals), no significant difference was found (paired Student's t-Test). This result might be due to the small amount of comparisons and/or to the presence of more true chimerical sequences present in the genome of hexaploid individuals.

## Discussion

Our phylogenetic analysis revealed *TOPO6* to be a single-copy locus in most diploid *Hordeum* species, as we found regularly only two different sequences per diploid individual, which we interpret as allelic variation. In a few cases we detected more than the expected maximum of two, four and six alleles in diploid, tetraploid and hexaploid individuals, respectively. However, except for three surprising cases (*H. murinum* H00219 and PI218078 and *H. bulbosum* BCC2018), these were very similar to each other so that we cannot safely discern if they stem from gene duplication or are artifacts originating during DNA amplification and sequencing. However, as using a proof-reading DNA polymerase in PCR greatly reduced the number of such sequence polymorphisms within individuals (Fig. S1) we assume that these are essentially PCR artifacts.

The phylogenetic relationships obtained from *TOPO6* are mainly congruent with previous work (reviewed in Blattner [9]) although they deviate from the total evidence phylogenetic tree of *Hordeum* [9]. This is not an unexpected result, as up to now no single marker region was able to ‘correctly’ resolve all species relationships among diploid *Hordeum* species [9], [34]. The major difference (Fig. 1) is the non-monophyly of subgenus *Hordeum* due to *H. murinum* being sister to subgenus *Hordeastrum* instead of the **H**-genome taxa, yet with weak statistical support. Minor differences, although no incompatibilities, concern the positions of (*i*) *H. bogdanii* and (*ii*) *H. californicum*, which group in the large polytomy of the New World species instead of *H. bogdanii* being sister to *H. californicum* plus the clade of the mainly South American taxa, and (*iii*) the non-monophyly of sequences derived from the three closely related Patagonian species *H. comosum*, *H. patagonicum*, and *H. pubiflorum*. Sequences of these species also group along the large polytomy in Figure 1, providing no hard contradiction to monophyly of this group. As the *TOPO6* sequences derived from polyploids mostly cluster with sequences obtained from specific diploid species in phylogenetic analyses, they enable the identification of the parental species involved in polyploid formation, which is the major goal of this study.

### Inference of extinct diploid progenitors of allopolyploids

Four statistically well-supported and genetically distinct clades in the phylogenetic trees contain only sequences derived from polyploid species but lack sequences of a currently existing diploid taxon: (*i*) in *H. murinum* (1.0 pp), (*ii*) in *H. gussoneanum* (1.0 pp), (*iii*) for *H. capense/H. secalinum* in the **I**-genome group (1.0 pp), and (*iv*) within the *H. californicum* group (1.0 pp). As we included all taxa of *Hordeum* in this study, mostly with multiple individuals representing the distribution areas of the species, we can safely interpret these sequences as the footprints of diploid species, which contributed their genome to allopolyploid taxa in the past and went extinct sometime after polyploid formation [35]. These data support similar findings from ITS and *EF-G* sequences for *H. murinum* [12], [14], [36] and ITS sequences for *H. californicum*-related taxa [14], *EF-G* data for tetraploid *H. gussoneanum* [37], and are compatible with the results of analyses of the *HTL* gene [38] and ITS [14] in *H. gussoneanum*. For *H. capense/H. secalinum* the identification of an extinct diploid progenitor in addition to *H. gussoneanum* is new. Based on the position of the *TOPO6* sequences between the clades formed by diploid *H. brevisubulatum* and *H. roshevitzii* (Fig. 2) we conclude that this extinct taxon belonged to the Central Asian group of *Hordeum* species, i.e. series *Sibirica*. Extinct progenitors of allopolyploids have been previously inferred [14], [35], [39]–[41]. However, the extent of this phenomenon is currently unclear, as the inclusion of single or few individuals as representatives of species is still common in phylogenetic studies (e.g., [13], [34]). This does not allow for the discerning of closely related species from conspecific individuals with high intraspecific genetic variation. Thus, Petersen and Seberg [10] obtained one type of *DMC1* sequences found in *H. capense* and *H. secalinum* as sistergroup of *H. brevisubulatum* while sequences of the second type grouped in a polytomy together with the sequence of *H. gussoneanum*. Accordingly, they interpreted this topology as an indication for *H. gussoneanum* and *H. brevisubulatum* being the parents of the tetraploids. The strong differentiation between their *H. capense/H. secalinum* and *H. brevisubulatum* sequences in *DMC1* are, however, completely compatible with the scenario we propose here, i.e. that the genepools of diploid *H. brevisubulatum* and the extinct progenitor of *H. capense/H. secalinum* belonged to separate taxa in the past. Particularly as neither *DMC1* nor *TOPO6* showed a comparably large differentiation for the tetraploid's sequences derived from *H. gussoneanum*, which should be the case if differentiation of the homoeologues evolved only after polyploid formation.

Due to insufficient state of data on such extinct species it is currently not possible to infer if extinction rates for diploids generally rise after they contribute to polyploid formation and, thus, increase competition in their habitats [42] or if we see the normal rate of background extinction in *Hordeum*. The ratio of 3∶1 of Old versus New World extinct species maintained in polyploids fits with the proposed generally higher Pleistocene extinction rates in Eurasia in comparison to the Americas inferred from the distribution of missing chloroplast haplotypes in *Hordeum* clades [43].

### Polyploid species of the Old World

In *H. bulbosum*, occurring in the Mediterranean and adjacent Southwest Asia, two cytotypes (2×, 4×) exist. All sequences but one (BCC2018_A) derived from this species are quite similar and group in a single strongly supported clade (1.0 pp), while the single outlier is sister to this group. For the diploid individuals we found, as expected, up to two *TOPO6* alleles and also in the tetraploid no more than two alleles were detected. Finding only one kind of sequence in a tetraploid could result from the loss of one copy from the genome, gene conversion, PCR-drift, limited clone sampling or autopolyploidy. As we included multiple individuals of the tetraploid in our study we do not expect that all would behave in the same way regarding technical shortcomings. Thus, our result supports the long-standing assumption that the tetraploid cytotype of *H. bulbosum* is of autopolyploid origin [44]. The peculiar position of the second type (A) of individual BCC2018 could indicate ancient introgression and/or incomplete lineage sorting [43] or pseudogenization of one *TOPO6* copy.

In the *H. murinum* taxon group two clades of *TOPO6* sequences were obtained. As already inferred by Jakob and Blattner [12] and Tanno et al. [36] in detailed studies of these taxa and cytotypes all polyploids are of allopolyploid origin involving extant and extinct species from within the **Xu**-genome group.

As discussed before, in the **Xa**-genome clade sequences obtained from *H. marinum* and *H. gussoneanum* (2×, 4×) formed three distinct clusters. The two copies of the tetraploid cytotype appeared in two of these clusters, one being exclusive to this cytotype (type A), which we interpret as an indication for an extinct diploid progenitor. Although *H. gussoneanum* was also involved in the evolution of three allopolyploid taxa combining **I** and **Xa** genomes, only extant *H. gussoneanum* contributed in these cases. One group of *TOPO6* sequences of the two tetraploid sister species *H. capense* and *H. secalinum* was clustering with the sequences derived from diploid *H. gussoneanum*, pinpointing this taxon as one parental species and are thus in accord with previous analyses [10], [14], [45], [46]. We found, however, no indications for a contribution of *H. marinum* to these tetraploids, as proposed by Taketa et al. [47] based on cytogenetic and Jakob and Blattner [43] on chloroplast data. Our data also confirm that *H. capense* and *H. secalinum* are very closely related, contrarily to Baum and Johnson [48], but clearly separated taxa, which most probably speciated after long-distance dispersal of *H. secalinum* from Europe to South Africa resulting in the geographically isolated *H. capense* [46].

Diploid *H. gussoneanum* introduced into North America contributed to the formation of the hexaploid cytotype of *H. brachyantherum* (haploid genome composition **IIXa**) via hybridization with *H. brachyantherum*, most probably in historic times. This result confirms previous observations [11], [14], [43], [45]. Our *TOPO6* sequences indicate that *H. gussoneanum* individuals originating from Spain (BCC2005) might have formed the Californian population (JB157) and contributed to *H. brachyantherum* (6×), occurring only in the Californian Bay area.

### *Hordeum roshevitzii*, a key species in the evolution of tetraploid *Hordeum* species

In addition to polyploid Central Asian *H. brevisubulatum* (discussed in detail below) all American tetraploid species with the exception of *H. depressum*, viz. *H. brachyantherum*, *H. fuegianum*, *H. guatemalense*, *H. jubatum*, and *H. tetraploidum*, carry one *TOPO6* type grouping with the sequences of *H. roshevitzii*, a diploid species endemic to Central Asia. The second sequence type derived from these species clustered with different diploid species of the American clade. North American *H. brachyantherum* and *H. jubatum* and Central American *H. guatemalense* thus have the *roshevitzii*-like *TOPO6* copy plus one of the extinct species identified within the *H. californicum* clade. In contrast to results from an ITS analysis [14] we found no indication for introgression of *H. intercedens* in *H. guatemalense*. In South American *H. tetraploidum* and *H. fuegianum*, in addition to the *roshevitzii*-like type, *TOPO6* copies close to that of the three Patagonian species *H. comosum/H. patagonicum/H. pubiflorum* occur. For *H. tetraploidum*, the sequences seem to hint towards a polyphyletic origin, as a group of individuals possess a different *TOPO6* copy derived from *H. pubiflorum*. From our data we cannot discern if *H. fuegianum* evolved from *H. tetraploidum* through a speciation event on the tetraploid level or if both taxa evolved through independent allopolyploidization involving the same parental species. To resolve this we would need a higher number of informative characters in DNA sequences and much more individuals of both species included in an analysis.

Overall, *H. roshevitzii* appears as the key species in the evolution of the tetraploid species of the New World. Our data confirm findings of Blattner [14] using ITS, where *H. roshevitzii* formed a clade with American allopolyploid species, and also cytogenetic data (FISH) that infer a contribution of *H. roshevitzii* to American allopolyploids [49]. Surprisingly, Wang and Sun [13], based on *DMC1* sequences, did not find a contribution of *H. roshevitzii* to American polyploid *Hordeum* taxa. Instead they detected in some American tetraploid and hexaploid species (*H. jubatum*, *H. fuegianum*, *H. tetraploidum*, and *H. arizonicum*) *DMC1* copies with close relationships to other Triticeae genera (*Taeniatherum* and *Pseudoroegneria*). The use of single individuals as representatives for taxa in this study makes it impossible to infer reasons for these differences, i.e. if this is a general feature of the polyploids or a peculiarity of the *DMC1* locus for single individuals.

In a geographic context the occurrence of *roshevitzii*-like sequences in North American polyploids indicates a second colonization event from Asia to North America after the initial establishment of *bogdanii*-like *Hordeum* diploids on that continent [46]. The *roshevitzii*-like sequences of the two South American tetraploids *H. tetraploidum* and *H. fuegianum* require either initial colonization of South America by *H. roshevitzii* and its later extinction after the formation of the tetraploids or the introduction of this sequence types through a polyploid (*H. jubatum*, see below) and introgression of the alleles via hybridization.

### The extinct *californicum*-like taxon was a key species in the evolution of American polyploids

The extinct close relative of *H. californicum*, to which we refer informally as *H. humatio*-*californicum* (due to the fact that it is closely related to *H. californicum* but that its genome today can only be found ‘buried’ in polyploid taxa), contributed its genome to tetraploid *H. brachyantherum* and *H. jubatum* and all New World hexaploids, i.e. *H. arizonicum* from North America and South American *H. lechleri*, *H. parodii* and *H. procerum*. Therefore, its importance for the evolution of *Hordeum* polyploids is comparable to that of *H. roshevitzii*. The American hexaploids all show essentially the *TOPO6* homoeolog pattern of tetraploid *H. jubatum*. In addition each hexaploid has a third copy clustering with American diploid species. Thus, the formation of these hexaploids can be explained by hybridization of *H. jubatum* with (*i*) diploid *H. pusillum* resulting in *H. arizonicum*, with (*ii*) *H. cordobense* resulting in *H. procerum*, with (*iii*) *H. pubiflorum* resulting in *H. lechleri*, and with (*iv*) *H. flexuosum* or, geographically less likely, *H. chilense* resulting in *H. parodii*. While the first two combinations seem plausible regarding the partly overlapping distribution areas of the species involved, and the third also for the overall high morphological similarity of *H. lechleri* with its proposed progenitors, in *H. parodii* we found only one individual with the *H. humatio-californicum* sequence, whereas four individuals possess a *TOPO6* copy related to *H. comosum/H. patagonicum/H. pubiflorum*. In this latter case, it might also be that *H. tetraploidum* instead of *H. jubatum* contributed to hexaploid formation and later-on introgression with *H. jubatum* or one of the other hexaploids contributed to additional genomic diversity. Alternatively, also a polyphyletic origin of *H. parodii* is compatible with the data. Chloroplast sequences do not contribute to the clarification of this topic, as apart from *H. lechleri*, all South American hexaploids possess chloroplast haplotypes derived from their South American diploid progenitors, i.e. *H. parodii* chloroplast haplotypes are shared with diploid *H. patagonicum* and *H. pubiflorum* and tetraploid *H. tetraploidum* [43].

In any case we have to assume that *H. jubatum* was present in South America for a long enough time to allow the evolution of at least three hexaploid taxa and their expansion to their extant partly very large and mainly allopatric distribution areas. This is in contradiction to the present assumption of a natural distribution of *H. jubatum* only in northwestern North America and northeastern Siberia [8] and its introduction into other areas of the world as ornamental in historic times. From the data obtained from the South American polyploids we infer the natural occurrence of *H. jubatum* in southern South America prior to European settlements and trade routes in this area. Thus, the scattered stands of this taxon in the grasslands of Central Argentina might well have originated by ancient bird-mediated [46] long-distance dispersal of this taxon from North America [43].

### *Hordeum depressum*, an All-American tetraploid

*Hordeum depressum* possesses a *TOPO6* copy (A) derived from *H. californicum* and a copy (B) clustering with diploid *H. intercedens* and *H. euclaston*. As it obtained no *roshevitzii*-like *TOPO6* copy it is the only ‘purely’ American tetraploid species. *Hordeum intercedens* from southwestern California and adjacent Mexico phylogenetically groups within South American *H. euclaston* [50]. Pronounced ecological differentiation [12] together with the geographically caused de facto reproductive isolation [46], [50] warrants the recognition as two independently evolving lineages, i.e. separate species. In contrast to Wang and Sun [13] who assumed that *H. euclaston* is a parent of *H. depressum*, we propose that in the frame of the geographical co-occurrence of *H. californicum* and *H. intercedens* in southern California these species are much more likely to hybridize and thus to have contributed to the evolution of the tetraploid.

### Hordeum brevisubulatum, a complex group

The taxon complex of *H. brevisubulatum* comprises five described subspecies from a large geographic area reaching from western Turkey to northeastern China with diploid, tetraploid and hexaploid cytotypes. Identification of the different subspecies is delicate especially for herbarium samples and materials from areas where subspecies overlap, thus we decided to consider only the ploidy level and the country of origin. The species is assumed to be of autopolyploid origin [14]. We were able to discern two major groups, with Iranian individuals found to be molecularly different from individuals from the rest of the species' distribution area. With one exception (H00312) the tetraploid individuals of *H. brevisubulatum* originating from Iran appeared to be autopolyploid, as the *TOPO6* sequences derived from these individuals clustered with the diploids only. Surprisingly, polyploid individuals originating from Siberia did not have a diploid *brevisubulatum*-like copy but instead one type of sequences derived from these eastern individuals clustered with *H. roshevitzii*. The other copy was either found in a peculiar position on a polytomy with the clades formed by **Xa** and **I**-genome sequences (BG156_07) or in the New World clade. In this latter clade they cluster together with sequences recovered from a tetraploid accession from Kirgizstan and a Tajik individual. For the hexaploid cytotypes, the individuals from Iran had a diploid *brevisubulatum*-like copy and the other one was clustering in the New World clade together with other *H. brevisubulatum* sequences. The hexaploid Tajik accession (BCC2016) had one sequence falling in the *H. roshevitzii* clade and one in the tetraploid/hexaploid *H. brevisubulatum* clade embedded in the New World clade. The *H. brevisubulatum* sequences present in this latter clade are similar to *H. bogdanii*, especially regarding the absence of the 33 bp long deletion characteristic for the American taxa.

To summarize our findings regarding *H. brevisubulatum*, according to the *TOPO6* phylogeny, only the Iranian *H. brevisubulatum* tetraploids are of autopolyploid origin, while in the remaining distribution area allopolyploids occur, which do not even include the diploid's *TOPO6* type. This species complex thus seems polyphyletic and/or exhibits signs of long-term interspecific hybridization with the three diploid Asian taxa *H. brevisubulatum*, *H. bogdanii* and *H. roshevitzii*. Moreover, *H. brevisubulatum* is the only species having one diploid individual with a *TOPO6* sequence clustering with the outgroup *Eremopyrum triticeum*, indicating introgression from outside of *Hordeum* [13], [51]. What we cannot estimate is if the genetic diversity found here represents the natural state of the species' lineages or if hybridization occurred during reproductive cycles in germplasm repositories. Obligate outbreeding of *H. brevisubulatum* [8] might facilitate introgression in comparison to most other *Hordeum* species.

### PCR-recombination results in chimerical sequences

Conducting this study, we tested for the influence of two different DNA polymerases on the proportion of chimerical sequences, combining parts of different *TOPO6* types occurring in *Hordeum*. Lahr and Katz [52] found that for genes of the major histocompatibility complex proof-reading DNA polymerases greatly reduce the proportion of recombinant sequences originating during PCR. In *Hordeum* we found the same effect, however, it seemed to vary according to the ploidy level, i.e. in tetraploid individuals the reduction of chimerical amplicons was much higher than in hexaploids. There are two possible explanations for this result. The first reason may be due to a lack of statistical power, as fewer comparisons were conducted (we did not increase the amount of screened colonies for hexa- in comparison to tetraploids). The second might be due to biological reasons, as recombinant copies might already be present in the genome. Cronn et al. [53] also found recombinant sequences of low-copy number genes in allopolyploid cotton species, which indicates that this phenomenon is not restricted to *Hordeum*. Although low-copy nuclear genes are very promising to reconstruct species phylogenies especially in polyploids [54], [55], the problem of recombinant sequences limits the usage of such markers. The origin of chimerical sequences during PCR amplification seems inherent to the type of sequences and to the use of universal primers, as they uniformly amplify all homoeologues, resulting in a mixture of amplicons. To minimize this problem one can design homoeolog-specific primers [10], [41], [53]. This means, however, that sequence information for the homoeologues has to be present in advance and prevents naïve exploring of allele diversity. Another solution, using single-molecule (sm) PCR [56], was employed by Marcussen et al. [57] to disentangle reticulate evolution in *Viola*. The smPCR seems particularly suitable for high-polyploid species and reduced sampling size. Nevertheless, from our experience in *Hordeum* we suggest that the use of a proof-reading DNA polymerase, probably together with low initial DNA concentration [52] in PCR, can reduce potential ambiguities regarding artificial recombinant amplicons. Although, we are well aware that this cannot supersede a careful inspection of the data, particularly when higher ploidy levels are involved.

### Conclusions

Using cloned sequences of *TOPO6*, a low-copy nuclear region, in a comprehensive framework including all *Hordeum* species mostly with several individuals, covering the geographic distribution of the species, we were able to infer parental relationships of *Hordeum* polyploids. The phylogenetic hypothesis presented here (Fig. 3) brought several new insights and supported other earlier data. Thus, it is likely that a close relative of Asian *H. bogdanii* was the starting point for the evolution of American diploid species. Diploid *H. roshevitzii* together with an extinct close relative of *H. californicum* and tetraploid *H. jubatum* were pivotal species for the evolution of the *Hordeum* allopolyploids. The involvement of *H. jubatum* in the formation of South American polyploids necessitates the presence of this taxon in South America well before the onset of European settlement. Thus we propose that also Central Argentina belongs to the natural distribution area of this species, resulting in a disjunct Northern–Southern Hemisphere distribution of *H. jubatum*. We were able to analyze the status of two putative autopolyploids, confirming autopolyploidy for *H. bulbosum* and tetraploid Iranian populations of *H. brevisubulatum*, while the latter taxon shows otherwise very complex and still poorly understood allopolyploid patterns. This species complex as well as some polyploids in South America might result from multiple independent origins or had a long history of hybridization and introgression. The use of proof-reading DNA polymerase in PCR can reduce phylogenetic noise when analyzing polyploid sequences by cloning. More in-depth analyses have to be performed to resolve the still unclear parental relationship, for example in some cases using more loci to arrive at a higher resolution for closely related species groups (in South America) or by including much more geographically representative individuals from natural populations for species complexes (particularly in *H. brevisubulatum*).

## Supporting Information

Figure S1 **Examples of chimeric** ***TOPO6*** **sequences together with non-recombinant sequences derived from a tetraploid individual of** ***Hordeum tetraploidum*** **(JB048C2B).** The striped alignment shows only polymorphic sites of all 16 clone-derived sequences (JB048C2Bb-q) and the two consensus sequences used in the analysis (*H. tetraploidum* JB048C2B_A and B). Sequences b–h were obtained using standard DNA polymerase and sequences i–q using proof-reading DNA polymerase in PCR. Sequences c, e, f and h show mosaic patterns indicated below the sequences as “A type” or “B type”, referring to the sequences used in the analysis. For this individual no chimerical sequences were retrieved with the proof-reading DNA polymerase against four obtained via regular DNA polymerase. Also the amount of singleton SNPs (PCR errors) found in the sequences differs between both polymerases.
(PDF)

Figure S2 **Strict consensus tree of 50,000 most parsimonious trees (L = 374 steps, CI = 0.84, RI = 0.96) from an analysis of** ***TOPO6*** **sequences derived from diploid** ***Hordeum*** **taxa and eight outgroup species.** Numbers along branches depict bootstrap values (%) of major clades of the tree derived from a ’fast-and-stepwise’ analysis of 50,000 bootstrap re-samples. *Psathyrostachys juncea* was defined as outgroup taxon in the analysis.
(PDF)

Figure S3 **Strict consensus tree of 50,000 most parsimonious trees (L = 536 steps, CI = 0.80, RI = 0.97) from an analysis of** ***TOPO6*** **sequences derived from di- and polyploid** ***Hordeum*** **taxa and eight outgroup species.** Numbers along branches depict bootstrap values (%) of major clades of the tree derived from a ‘fast-and-stepwise’ analysis of 50,000 bootstrap re-samples. *Psathyrostachys juncea* was defined as outgroup taxon in the analysis.
(PDF)

Figure S4 **Part of the** ***TOPO6*** **alignment showing a 33 bp deletion (alignment positions 925–957) synapomorphic for sequences derived from diploid New World** ***Hordeum*** **species.** Sequence deletion occurred at a five basepair direct repeat (TACAC) flanking the deleted region (arrows). The absence of the deletion in *H. bogdanii* together with its presence in all American diploid species indicates that not *H. bogdanii* itself but a close relative of this species was the initial starting point for the colonization of the Americas by Asian *Hordeum*.
(PDF)

Table S1 **Detailed information for all individuals analyzed in this study.**
(PDF)

File S1 **Alignment of di- and polyploid-derived** ***TOPO6*** **sequences.**
(FASTA)

File S2 **Alignment of diploid-derived** ***TOPO6*** **sequences.**
(FASTA)
