# Supplementary material for: Progenitor-Derivative Relationships of Hordeum Polyploids (Poaceae, Triticeae) Inferred from Sequences of TOPO6, a Nuclear Low-Copy Gene Region
Source: PLoS One. 2012 Mar 30;7(3):e33808. doi: 10.1371/journal.pone.0033808 (PMC3316500; doi:10.1371/journal.pone.0033808)
Supplement: Figure S1 — Examples of chimeric TOPO6 sequences together with non-recombinant sequences derived from a tetraploid individual of Hordeum tetraploidum (JB048C2B). The striped alignment shows only polymorphic sites of all 16 clone-derived sequences (JB048C2Bb-q) and the two consensus sequences used in the analysis (H. tetraploidum JB048C2B_A and B). Sequences b–h were obtained using standard DNA polymerase and sequences i–q using proof-reading DNA polymerase in PCR. Sequences c, e, f and h show mosaic patterns indicated below the sequences as “A type” or “B type”, referring to the sequences used in the analysis. For this individual no chimerical sequences were retrieved with the proof-reading DNA polymerase against four obtained via regular DNA polymerase. Also the amount of singleton SNPs (PCR errors) found in the sequences differs between both polymerases. (PDF) [file pone.0033808.s001.pdf]

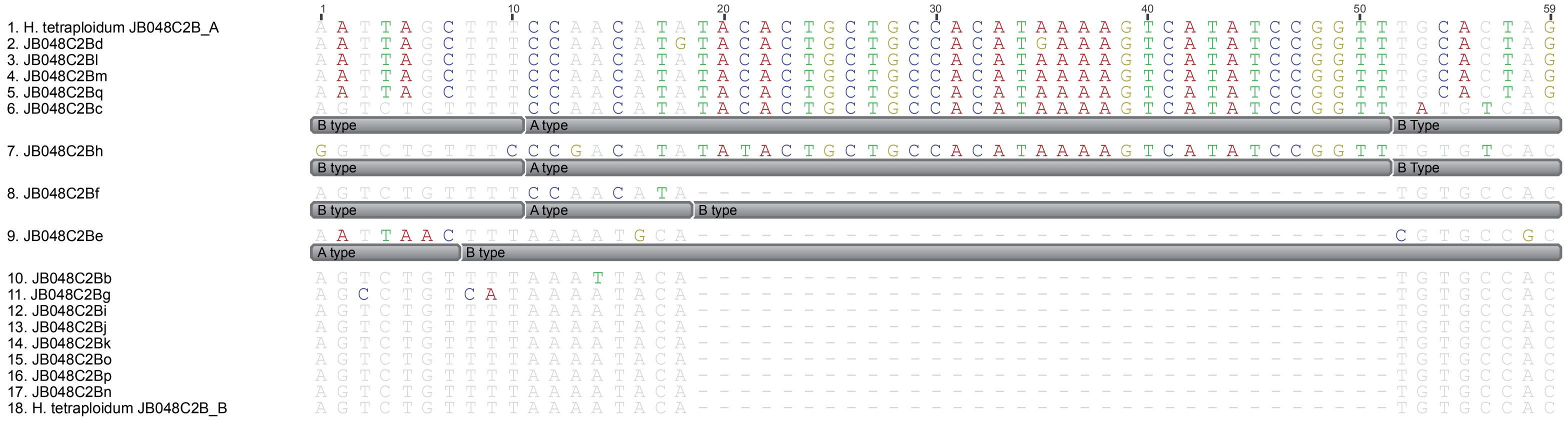

Figure S1 Examples of chimeric TOPO6 sequences together with non-recombinant sequences derived from a tetraploid individual of Hordeum tetraploidum (JB048C2B). The striped alignment shows only polymorphic sites of all 16 clone-derived sequenced (JB048C2Bb-q) and the two consensus sequences used in the analysis (H. tetraploidum JB048C2B_A and B). Sequences b–h were obtained using standard DNA polymerase and sequences i–q using proof-reading DNA polymerase in PCR. Sequences c, e, f and h show mosaic patterns indicated below the sequences as “A type” or “B type”, referring to the sequences used in the analysis. For this individual no chimerical sequences were retrieved with the proof-reading DNA polymerase against four obtained via regular DNA polymerase. Also the amount of singleton SNPs (PCR errors) found in the sequences differs between both polymerases.
