# Supplementary material for: Progenitor-Derivative Relationships of Hordeum Polyploids (Poaceae, Triticeae) Inferred from Sequences of TOPO6, a Nuclear Low-Copy Gene Region
Source: PLoS One. 2012 Mar 30;7(3):e33808. doi: 10.1371/journal.pone.0033808 (PMC3316500; doi:10.1371/journal.pone.0033808)
Supplement: Figure S2 — Strict consensus tree of 50,000 most parsimonious trees (L = 374 steps, CI = 0.84, RI = 0.96) from an analysis of TOPO6 sequences derived from diploid Hordeum taxa and eight outgroup species. Numbers along branches depict bootstrap values (%) of major clades of the tree derived from a ’fast-and-stepwise’ analysis of 50,000 bootstrap re-samples. Psathyrostachys juncea was defined as outgroup taxon in the analysis. (PDF) [file pone.0033808.s002.pdf]

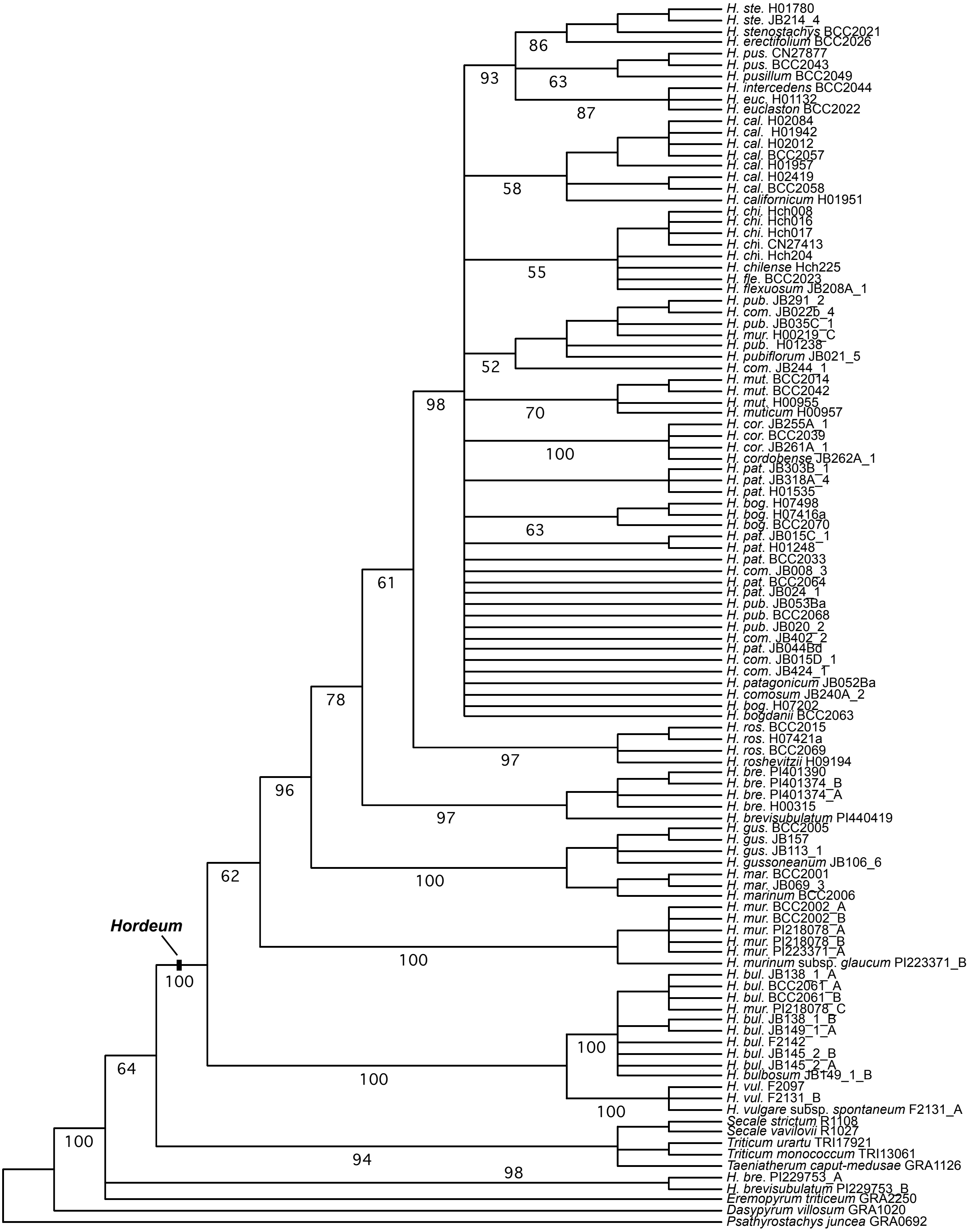

Figure S2. Strict consensus tree of 50,000 most parsimonious trees (L = 374 steps, CI = 0.84, RI = 0.96) from an analysis of TOPO6 sequences derived from diploid *Hordeum* taxa and eight outgroup species. Numbers along branches depict bootstrap values (%) of major clades of the tree derived from a 'fast-and-stepwise' analysis of 50,000 bootstrap re-samples. *Psathyrostachys juncea* was defined as outgroup taxon in the analysis.
