# Supplementary figures and images for: Progenitor-Derivative Relationships of Hordeum Polyploids (Poaceae, Triticeae) Inferred from Sequences of TOPO6, a Nuclear Low-Copy Gene Region
Source: PLoS One. 2012 Mar 30;7(3):e33808. doi: 10.1371/journal.pone.0033808 (PMC3316500; doi:10.1371/journal.pone.0033808)

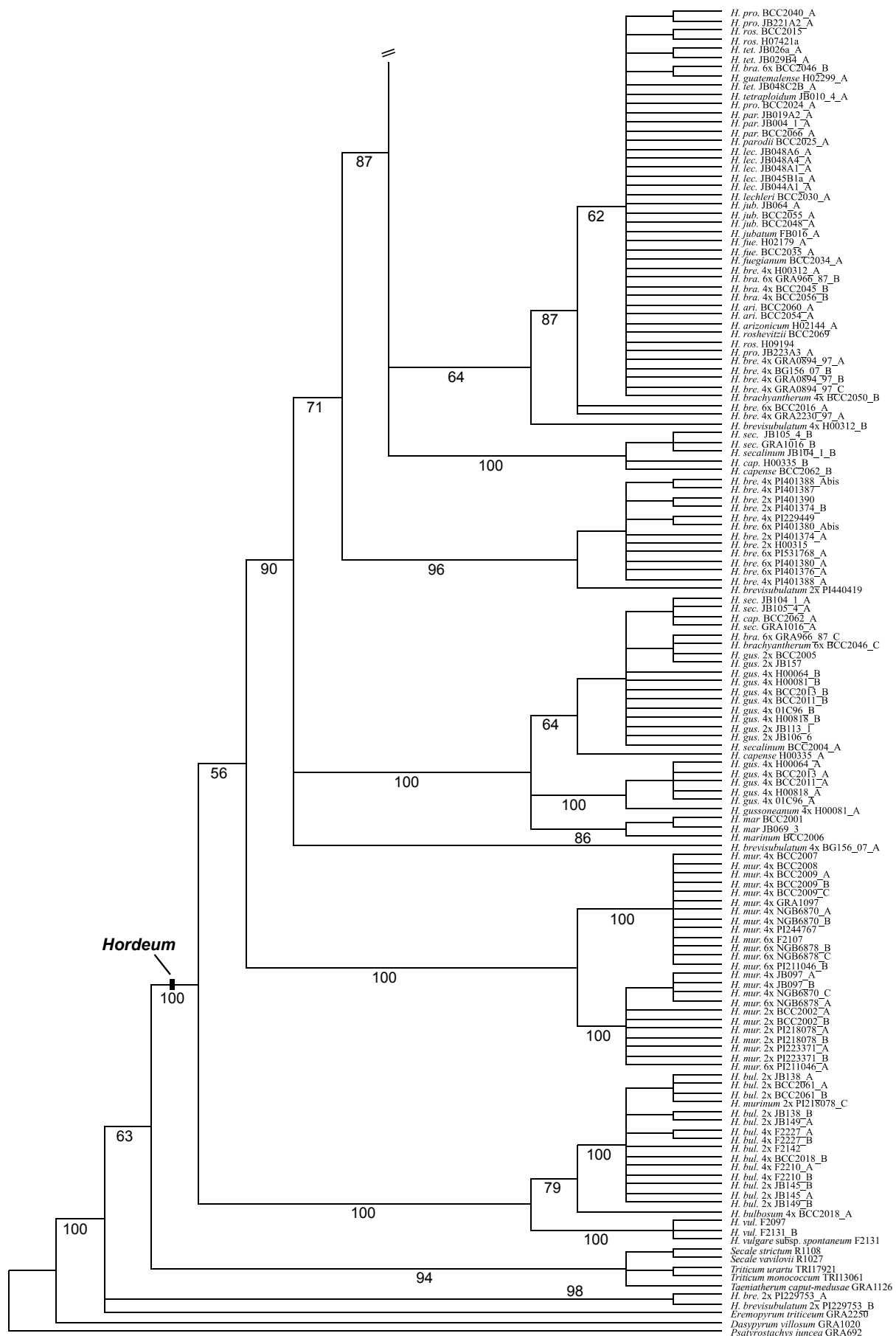

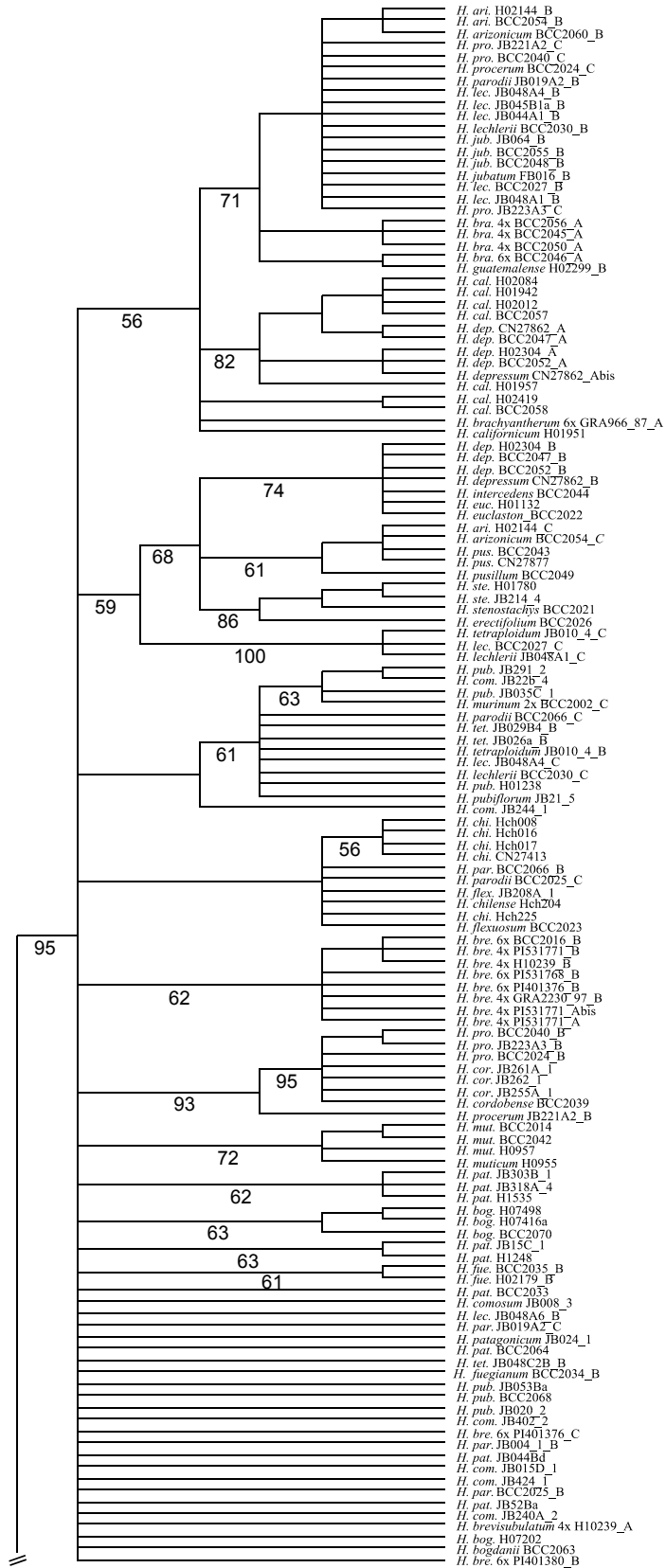

Figure S3. (Continued)

Supplement: Figure S3 — Strict consensus tree of 50,000 most parsimonious trees (L = 536 steps, CI = 0.80, RI = 0.97) from an analysis of TOPO6 sequences derived from di- and polyploid Hordeum taxa and eight outgroup species. Numbers along branches depict bootstrap values (%) of major clades of the tree derived from a ‘fast-and-stepwise’ analysis of 50,000 bootstrap re-samples. Psathyrostachys juncea was defined as outgroup taxon in the analysis. (PDF) [file pone.0033808.s003.pdf]
