# Supplementary material for: Progenitor-Derivative Relationships of Hordeum Polyploids (Poaceae, Triticeae) Inferred from Sequences of TOPO6, a Nuclear Low-Copy Gene Region
Source: PLoS One. 2012 Mar 30;7(3):e33808. doi: 10.1371/journal.pone.0033808 (PMC3316500; doi:10.1371/journal.pone.0033808)
Supplement: Figure S4 — Part of the TOPO6 alignment showing a 33 bp deletion (alignment positions 925–957) synapomorphic for sequences derived from diploid New World Hordeum species. Sequence deletion occurred at a five basepair direct repeat (TACAC) flanking the deleted region (arrows). The absence of the deletion in H. bogdanii together with its presence in all American diploid species indicates that not H. bogdanii itself but a close relative of this species was the initial starting point for the colonization of the Americas by Asian Hordeum. (PDF) [file pone.0033808.s004.pdf]

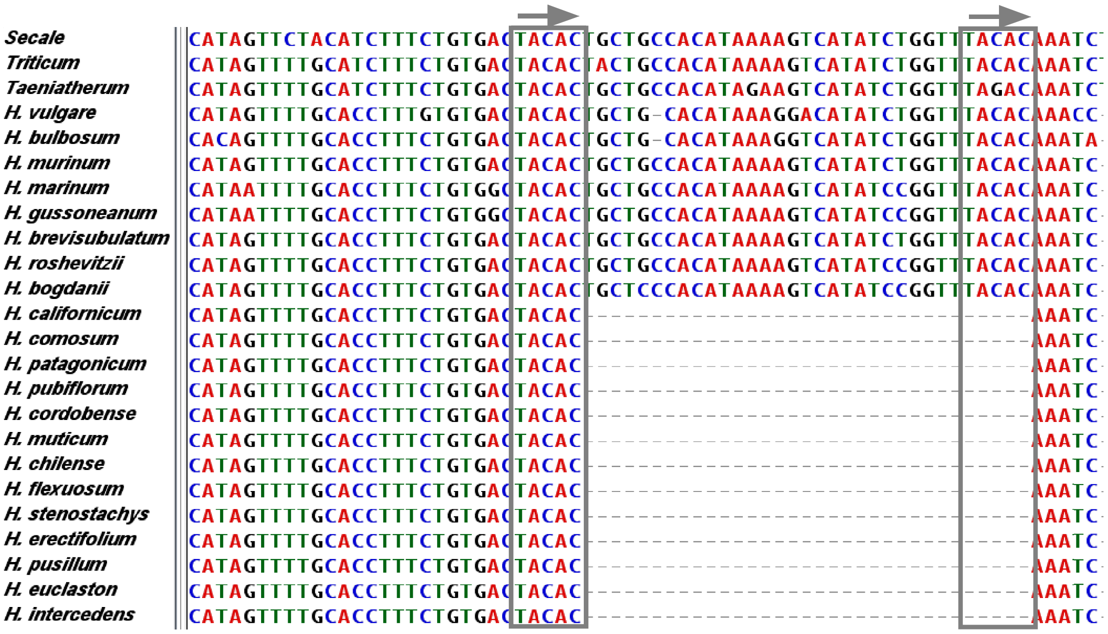

Figure S4. Part of the *TOPO6* alignment showing a 33 bp deletion (alignment positions 925-957) characteristic for sequences derived from diploid New World *Hordeum* species. Sequence deletion occurred at a five basepair direct repeat (TACAC) flanking the deleted region (arrows). The absence of the deletion in *H. bogdanii* together with its presence in all American diploid species indicates that not *H. bogdanii* itself but a close relative of this species was the initial starting point for the colonization of the Americas by Asian *Hordeum*.
