# Supplementary material for: Progenitor-Derivative Relationships of Hordeum Polyploids (Poaceae, Triticeae) Inferred from Sequences of TOPO6, a Nuclear Low-Copy Gene Region
Source: PLoS One. 2012 Mar 30;7(3):e33808. doi: 10.1371/journal.pone.0033808 (PMC3316500; doi:10.1371/journal.pone.0033808)
Supplement: Table S1 — Detailed information for all individuals analyzed in this study. (PDF) [file pone.0033808.s005.pdf]

Table S1 Individuals included in the study

Species	Ploidy	Accession number	Country	Species distribution	Material source	Topo6 type
Hordeum arizonicum Covas	6x	BCC2054 (H02313)	USA	North America	Barley Core Collection	*H. arizonicum* BCC2054_A, B, C
		BCC2060 (H03253)	USA	North America	Barley Core Collection	*H. arizonicum* BCC2060_A, B
		H02144 (NGB90553)	Mexico	North America	Nordic Genetic Resource Centre	*H. arizonicum* H02144_A, B, C
Hordeum bogdanii Wilensky	2x	BCC2070 (H07804)	China	Central Asia	Barley Core Collection	*H. bogdanii* BCC2070
		H07065 (NGB06797)	China	Central Asia	Nordic Genetic Resource Centre	
		H07421b (NGB07634)	China	Central Asia	Nordic Genetic Resource Centre	
		H07429 (NGB06893)	China	Central Asia	Nordic Genetic Resource Centre	
		H07436 (NGB90003)	China	Central Asia	Nordic Genetic Resource Centre	
		H07461 (NGB07281)	China	Central Asia	Nordic Genetic Resource Centre	
		H07465 (NGB90048)	China	Central Asia	Nordic Genetic Resource Centre	
		H07476 (NGB08522)	China	Central Asia	Nordic Genetic Resource Centre	
		H07485b (NGB07282)	China	Central Asia	Nordic Genetic Resource Centre	
		H07569 (NGB08525)	China	Central Asia	Nordic Genetic Resource Centre	
		H07752 (NGB90004)	China	Central Asia	Nordic Genetic Resource Centre	
		H07831 (NGB90006)	China	Central Asia	Nordic Genetic Resource Centre	
		H07859 (NGB90007)	China	Central Asia	Nordic Genetic Resource Centre	
		H08704 (NGB90232)	China	Central Asia	Nordic Genetic Resource Centre	
		H08774 (NGB90049)	China	Central Asia	Nordic Genetic Resource Centre	
		H09215 (NGB90548)	China	Central Asia	Nordic Genetic Resource Centre	
		H03084 (NGB90002)	China	Central Asia	Nordic Genetic Resource Centre	
		PI499499	China	Central Asia	GRIN USDA Aberdeen, USA	
		PI499500	China	Central Asia	GRIN USDA Aberdeen, USA	
		PI531760	China	Central Asia	GRIN USDA Aberdeen, USA	
		BCC2063 (H04014)	Pakistan	Central Asia	Barley Core Collection	*H. bogdanii* BCC2063
		GRA0969	Pakistan	Central Asia	Gene bank IPK	
		H00240 (NGB08519)	Afghanistan	Central Asia	Nordic Genetic Resource Centre	
		H00295 (NGB06440)	Pakistan	Central Asia	Nordic Genetic Resource Centre	
		H07067 (NGB06798)	China	Central Asia	Nordic Genetic Resource Centre	
		H07411 (NGB90229)	China	Central Asia	Nordic Genetic Resource Centre	
		H08769 (NGB90337)	China	Central Asia	Nordic Genetic Resource Centre	
		H09213 (NGB90547)	China	Central Asia	Nordic Genetic Resource Centre	
		PI499501	China	Central Asia	GRIN USDA Aberdeen, USA	
		PI499646	China	Central Asia	GRIN USDA Aberdeen, USA	
		H08700 (NGB90231)	China	Central Asia	Nordic Genetic Resource Centre	
		H00287 (NGB6439)	Pakistan	Central Asia	Nordic Genetic Resource Centre	
		H07498 (NGB08523)	China	Central Asia	Nordic Genetic Resource Centre	*H. bogdanii* H07498
		H07521 (NGB90047)	China	Central Asia	Nordic Genetic Resource Centre	
		H07557 (NGB08524)	China	Central Asia	Nordic Genetic Resource Centre	
		H07585 (NGB07283)	China	Central Asia	Nordic Genetic Resource Centre	
		H07727 (NGB90230)	China	Central Asia	Nordic Genetic Resource Centre	
		PI499498	China	Central Asia	GRIN USDA Aberdeen, USA	
		PI531761	China	Central Asia	GRIN USDA Aberdeen, USA	
		H07202 (NGB90385)	China	Central Asia	Nordic Genetic Resource Centre	*H. bogdanii* H07202
		H07416a (NGB08586)	China	Central Asia	Nordic Genetic Resource Centre	*H. bogdanii* H07416a
Hordeum brachyantherum Nevski	4x	BCC2045 (H01958)	USA	North America	Barley Core Collection	*H. brachyantherum* 4x BCC2045_A, B
		BCC2050 (H02138)	USA	North America	Barley Core Collection	*H. brachyantherum* 4x BCC2050_A, B
		BCC2056 (H02360)	Canada	North America	Barley Core Collection	*H. brachyantherum* 4x BCC2056_A, B
	6x	BCC2046 (H02001, GRA0968)	USA	North America	Barley Core Collection	*H. brachyantherum* 6x BCC2046_A, B, C
		GRA0966/87	USA	North America	Gene bank IPK	*H. brachyantherum* 6x GRA0966_87_A, B, C
Hordeum brevisubulatum (Trin.) Link	2x	PI229753	Iran	Central Asia	GRIN USDA Aberdeen, USA	*H. brevisubulatum* 2x PI229753_A, B
		PI401374	Iran	Central Asia	GRIN USDA Aberdeen, USA	*H. brevisubulatum* 2x PI401374_A, B
		PI401390	Iran	Central Asia	GRIN USDA Aberdeen, USA	*H. brevisubulatum* 2x PI401390
		H00315	Iran	Central Asia	Nordic Genetic Resource Centre	*H. brevisubulatum* 2x H00315
		PI440419	Russia	Central Asia	GRIN USDA Aberdeen, USA	*H. brevisubulatum* 2x PI440419
	4x	H10239 (NGB90440)	Tajikistan	Central Asia	Nordic Genetic Resource Centre	*H. brevisubulatum* 4x H10239_A, B
		BG156/07	Russia	Central Asia	Barley Core Collection	*H. brevisubulatum* 4x BG156_07_A, B

**Species**	**Ploidy**	**Accession number**	**Country**	**Species distribution**	**Material source**	**Topo6 type**
		PI401387	Iran	Central Asia	GRIN USDA Aberdeen, USA	*H. brevisubulatum* 4x PI401387
		PI401388	Iran	Central Asia	GRIN USDA Aberdeen, USA	*H. brevisubulatum* 4x PI401388_A, Abis
		PI229449	Iran	Central Asia	GRIN USDA Aberdeen, USA	*H. brevisubulatum* 4x PI229449
		PI531771	Kyrgyzstan	Central Asia	GRIN USDA Aberdeen, USA	*H. brevisubulatum* 4x PI531771_A, Abis, B
	6x	BCC2016 (H10210)	Tajikistan	Central Asia	Barley Core Collection	*H. brevisubulatum* 6x BCC2016_A, B
		PI401376	Iran	Central Asia	GRIN USDA Aberdeen, USA	*H. brevisubulatum* 6x PI401376_A, B, C
		PI401380	Iran	Central Asia	GRIN USDA Aberdeen, USA	*H. brevisubulatum* 6x PI401380_A, Abis, B
		PI531768	Tajikistan	Central Asia	GRIN USDA Aberdeen, USA	*H. brevisubulatum* 6x PI531768_A, B
	4x	GRA2230/97	Russia	Central Asia	Gene bank IPK	*H. brevisubulatum* 4x GRA2230_97_A, B
		GRA0894/97	Russia	Central Asia	Gene bank IPK	*H. brevisubulatum* 4x GRA0894_97_A, B, C
		H00312 (NGB90046)	Iran	Central Asia	Nordic Genetic Resource Centre	*H. brevisubulatum* 4x H00312_A, B
Hordeum bulbosum L.	2x	BCC2061 (H03878)	Italy	Europe/SW Asia/Mediterranean	Barley Core Collection	*H. bulbosum* 2x BCC2061_A, B
		JB138_1	Italy	Europe/SW Asia/Mediterranean	Sabine Jakob, Frank Blattner	*H. bulbosum* 2x JB138_1_A, B
		JB145_2	Italy	Europe/SW Asia/Mediterranean	Sabine Jakob, Frank Blattner	*H. bulbosum* 2x JB145_2_A, B
		JB149_1	Italy	Europe/SW Asia/Mediterranean	Sabine Jakob, Frank Blattner	*H. bulbosum* 2x JB149_1_A, B
		F2142	Uzbekistan	Europe/SW Asia/Mediterranean	Reinhard Fritsch	*H. bulbosum* 2x F2142
	4x	F2210	Iran	Europe/SW Asia/Mediterranean	Reinhard Fritsch	*H. bulbosum* 4x F2210_A, B
		F2227	Iran	Europe/SW Asia/Mediterranean	Reinhard Fritsch	*H. bulbosum* 4x F2227_A, B
		BCC2018 (H10298)	Tajikistan	Europe/SW Asia/Mediterranean	Barley Core Collection	*H. bulbosum* 4x BCC2018_A, B
Hordeum californicum Covas & Stebbins	2x	H02419 (NGB90225)	USA	North America	Nordic Genetic Resource Centre	*H. californicum* H02419
		H02428 (NGB90227)	USA	North America	Nordic Genetic Resource Centre	
		H02445 (NGB90228)	USA	North America	Nordic Genetic Resource Centre	
		H02084 (NGB90009)	USA	North America	Nordic Genetic Resource Centre	*H. californicum* H02084
		H02408 (NGB90223)	USA	North America	Nordic Genetic Resource Centre	
		H01942 (NGB06800)	USA	North America	Nordic Genetic Resource Centre	*H. californicum* H01942
		H02423 (NGB90226)	USA	North America	Nordic Genetic Resource Centre	
		H02012 (NGB06462)	USA	North America	Nordic Genetic Resource Centre	*H. californicum* H02012
		H02414 (NGB90224)	USA	North America	Nordic Genetic Resource Centre	
		H01957 (NGB06802)	USA	North America	Nordic Genetic Resource Centre	*H. californicum* H01957
		BCC2057 (H02401)	USA	North America	Barley Core Collection	*H. californicum* BCC2057
		BCC2058 (H02428)	USA	North America	Barley Core Collection	*H. californicum* BCC2058
		H01951 (NGB06801)	USA	North America	Nordic Genetic Resource Centre	*H. californicum* H01951
Hordeum capense Thunb.	4x	BCC2062 (H03923)	South Africa	South Africa	Barley Core Collection	*H. capense* BCC2062_A, B
		H00335 (NGB90596)	Lesotho	South Africa	Nordic Genetic Resource Centre	*H. capense* H00335_A, B
Hordeum chilense Roemer & Schultes	2x	Hch204	Chile	South America	Pilar Hernandez	*H. chilense* Hch204
		Hch207	Chile	South America	Pilar Hernandez	
		Hch210	Chile	South America	Pilar Hernandez	
		Hch212	Chile	South America	Pilar Hernandez	
		Hch290	Chile	South America	Pilar Hernandez	
		Hch298	Chile	South America	Pilar Hernandez	
		Hch308	Chile	South America	Pilar Hernandez	
		JB006A_2	Argentina	South America	Sabine Jakob, Frank Blattner	
		JB006A_4	Argentina	South America	Sabine Jakob, Frank Blattner	
		JB006C_12	Argentina	South America	Sabine Jakob, Frank Blattner	
		JB006C_13	Argentina	South America	Sabine Jakob, Frank Blattner	
		Hch017	Chile	South America	Pilar Hernandez	*H. chilense* Hch017
		Hch068	Chile	South America	Pilar Hernandez	
		Hch245	Chile	South America	Pilar Hernandez	
		Hch250	Chile	South America	Pilar Hernandez	
		Hch261	Chile	South America	Pilar Hernandez	
		Hch300	Chile	South America	Pilar Hernandez	
		CN27413	Argentina	South America	AGR, Saskatoon, Canada	*H. chilense* CN27413
		Hch251	Chile	South America	Pilar Hernandez	
		Hch016	Chile	South America	Pilar Hernandez	*H. chilense* Hch016
		Hch225	Chile	South America	Pilar Hernandez	*H. chilense* Hch225
		Hch008	Chile	South America	Pilar Hernandez	*H. chilense* Hch008
Hordeum comosum Presl	2x	JB008_3	Argentina	South America	Sabine Jakob, Frank Blattner	*H. comosum* JB008_3
		JB307_1	Argentina	South America	Sabine Jakob, Frank Blattner	
		JB316A_1	Argentina	South America	Sabine Jakob, Frank Blattner	
		JB316A_3	Argentina	South America	Sabine Jakob, Frank Blattner	

Species	Ploidy	Accession number	Country	Species distribution	Material source	Topo6 type
		JB412_1	Argentina	South America	Sabine Jakob, Frank Blattner	
		JB413_1	Argentina	South America	Sabine Jakob, Frank Blattner	
		JB415_2	Argentina	South America	Sabine Jakob, Frank Blattner	
		JB418_1	Argentina	South America	Sabine Jakob, Frank Blattner	
		JB421B_4	Argentina	South America	Sabine Jakob, Frank Blattner	
		JB468_1	Argentina	South America	Sabine Jakob, Frank Blattner	
		JB015D_1	Argentina	South America	Sabine Jakob, Frank Blattner	*H. comosum* JB015D_1
		JB053C_3	Argentina	South America	Sabine Jakob, Frank Blattner	
		JB236A_2	Argentina	South America	Sabine Jakob, Frank Blattner	
		JB281A_1	Argentina	South America	Sabine Jakob, Frank Blattner	
		JB410_1	Argentina	South America	Sabine Jakob, Frank Blattner	
		JB447B_2	Argentina	South America	Sabine Jakob, Frank Blattner	
		JB448_3	Argentina	South America	Sabine Jakob, Frank Blattner	
		JB467_3	Argentina	South America	Sabine Jakob, Frank Blattner	
		JB402_2	Argentina	South America	Sabine Jakob, Frank Blattner	*H. comosum* JB402_2
		JB402_5	Argentina	South America	Sabine Jakob, Frank Blattner	
		JB022B_4	Argentina	South America	Sabine Jakob, Frank Blattner	*H. comosum* JB022B_4
		JB240A_2	Argentina	South America	Sabine Jakob, Frank Blattner	*H. comosum* JB240A_2
		JB244_1	Argentina	South America	Sabine Jakob, Frank Blattner	*H. comosum* JB244_1
		JB424_1	Argentina	South America	Sabine Jakob, Frank Blattner	*H. comosum* JB424_1
Hordeum cordobense Bothmer, Jacobsen & Nicora	2x	BCC2039 (H01702, GRA0974)	Argentina	South America	Barley Core Collection	*H. cordobense* BCC2039
		BCC2067 (H06429)	Argentina	South America	Barley Core Collection	
		JB247A_1	Argentina	South America	Sabine Jakob, Frank Blattner	
		JB247A_3	Argentina	South America	Sabine Jakob, Frank Blattner	
		JB249_1	Argentina	South America	Sabine Jakob, Frank Blattner	
		JB249_2	Argentina	South America	Sabine Jakob, Frank Blattner	
		JB249_3	Argentina	South America	Sabine Jakob, Frank Blattner	
		JB253A_1	Argentina	South America	Sabine Jakob, Frank Blattner	
		JB253A_2	Argentina	South America	Sabine Jakob, Frank Blattner	
		JB253A_3	Argentina	South America	Sabine Jakob, Frank Blattner	
		JB262_3	Argentina	South America	Sabine Jakob, Frank Blattner	
		JB263_1	Argentina	South America	Sabine Jakob, Frank Blattner	
		JB263_2	Argentina	South America	Sabine Jakob, Frank Blattner	
		JB265_1	Argentina	South America	Sabine Jakob, Frank Blattner	
		JB255A_1	Argentina	South America	Sabine Jakob, Frank Blattner	*H. cordobense* JB225A_1
		JB255A_2	Argentina	South America	Sabine Jakob, Frank Blattner	
		JB255A_3	Argentina	South America	Sabine Jakob, Frank Blattner	
		JB257A_1	Argentina	South America	Sabine Jakob, Frank Blattner	
		JB261A_1	Argentina	South America	Sabine Jakob, Frank Blattner	*H. cordobense* JB261A_1
		JB263_3	Argentina	South America	Sabine Jakob, Frank Blattner	
		JB262_1	Argentina	South America	Sabine Jakob, Frank Blattner	*H. cordobense* JB262_1
		JB262_2	Argentina	South America	Sabine Jakob, Frank Blattner	
Hordeum depressum (Scribn. & J. G. Sm.) Rydb.	4x	BCC2047 (H02006)	USA	North America	Barley Core Collection	*H. depressum* BCC2047_A, B
		BCC2052 (H02306)	USA	North America	Barley Core Collection	*H. depressum* BCC2052_A, B
		CN27862	USA	North America	AGR, Saskatoon, Canada	*H. depressum* CN27862_A, Abis, B
		H02304 (NGB06810)	USA	North America	Nordic Genetic Resource Centre	*H. depressum* H02304_A, B
Hordeum erectifolium Bothmer, Jacobsen & Jørgensen	2x	BCC2026 (H01150)	Argentina	South America	Barley Core Collection	*H. erectifolium* BCC2026
Hordeum euclaston Steud.	2x	BCC2022 (H01115)	Argentina	South America	Barley Core Collection	*H. euclaston* BCC2022
		BCC2029 (H01263)	Argentina	South America	Barley Core Collection	
		CN27340	Argentina	South America	AGR, Saskatoon, Canada	
		CN27343	Argentina	South America	AGR, Saskatoon, Canada	
		CN27359	Argentina	South America	AGR, Saskatoon, Canada	
		CN27369	Argentina	South America	AGR, Saskatoon, Canada	
		H01103 (NGB08534)	Argentina	South America	Nordic Genetic Resource Centre	
		H01107 (NGB06465)	Argentina	South America	Nordic Genetic Resource Centre	
		H02148 (NGB90233)	Uruguay	South America	Nordic Genetic Resource Centre	
		H06045 (NGB90342)	Argentina	South America	Nordic Genetic Resource Centre	
		JB225A	Argentina	South America	Sabine Jakob, Frank Blattner	
		JB228A_1	Argentina	South America	Sabine Jakob, Frank Blattner	
		JB228A_6	Argentina	South America	Sabine Jakob, Frank Blattner	
		H01132 (NGB07289)	Argentina	South America	Nordic Genetic Resource Centre	*H. euclaston* H01132
H. flexuosum Steud.		BCC2023 (H01133, NGB06470)	Argentina	South America	Nordic Genetic Resource Centre	*H. flexuosum* BCC2023
		CN27346	Argentina	South America	AGR, Saskatoon, Canada	

Species	Ploidy	Accession number	Country	Species distribution	Material source	Topo6 type
		CN27358	Argentina	South America	AGR, Saskatoon, Canada	
		JB208A_2	Argentina	South America	Sabine Jakob, Frank Blattner	
		JB208A_3	Argentina	South America	Sabine Jakob, Frank Blattner	
		JB208A_1	Argentina	South America	Sabine Jakob, Frank Blattner	*H. flexuosum* JB208A_1
		CN27348	Argentina	South America	AGR, Saskatoon, Canada	
		CN27399	Argentina	South America	AGR, Saskatoon, Canada	
Hordeum fuegianum Bothmer, Jacobsen & Jørgensen	4x	BCC2034 (H01371)	Argentina	South America	Barley Core Collection	*H. fuegianum* BCC2034_A, B
		BCC2035 (H01422)	Argentina	South America	Barley Core Collection	*H. fuegianum* BCC2035_A, B
		H02179 (NGB90017)	Chile	South America	Nordic Genetic Resource Centre	*H. fuegianum* H02179_A, B
Hordeum guatemalense Bothmer, Jacobsen & Jørgensen	4x	H02299 (NGB90554)	Guatemala	South America	Nordic Genetic Resource Centre	*H. guatemalense* H02299_A, B
Hordeum intercedens Nevski	2x	BCC2044 (H01940, GRA979)	USA	North America	Barley Core Collection	*H. intercedens* BCC2044
		BCC2059 (H03252)	Mexico	North America	Barley Core Collection	
		CN28638	Mexico	North America	AGR, Saskatoon, Canada	
		CN28639	Mexico	North America	AGR, Saskatoon, Canada	
		CN28640	Mexico	North America	AGR, Saskatoon, Canada	
		CN28643	Mexico	North America	AGR, Saskatoon, Canada	
		CN28644	Mexico	North America	AGR, Saskatoon, Canada	
Hordeum jubatum L.	4x	BCC2048 (H02018)	Mexico	North America	Barley Core Collection	*H. jubatum* BCC2048_A, B
		BCC2055 (H02324)	USA	North America	Barley Core Collection	*H. jubatum* BCC2055_A, B
		JB064	Germany	North America	Sabine Jakob, Frank Blattner	*H. jubatum* JB064_A, B
		FB016	Argentina	South America	Frank Blattner	*H. jubatum* FB016_A, B
Hordeum lechleri (Steud.) Schenck	6x	BCC2027 (H01185)	Argentina	South America	Barley Core Collection	*H. lechleri* BCC2027_B, C
		BCC2030 (H01310)	Argentina	South America	Barley Core Collection	*H. lechleri* BCC2030_A, B, C
		JB044A_1	Argentina	South America	Sabine Jakob, Frank Blattner	*H. lechleri* JB044A1_A, B
		JB048A_4	Argentina	South America	Sabine Jakob, Frank Blattner	*H. lechleri* JB048A4_A, B, C
		JB045B_1a	Argentina	South America	Sabine Jakob, Frank Blattner	*H. lechleri* JB045B1a_A, B
		JB048A_6	Argentina	South America	Sabine Jakob, Frank Blattner	*H. lechleri* JB048A6_A, B
		JB048A_1	Argentina	South America	Sabine Jakob, Frank Blattner	*H. lechleri* JB048A1_A, B, C
Hordeum gussoneanum Parl.	2x	BCC2005 (H00539, NGB7294)	Spain	Europe/SW Asia/Mediterranean	Barley Core Collection	*H. gussoneanum* 2x BCC2005
		JB106_6	France	Europe/SW Asia/Mediterranean	Sabine Jakob, Frank Blattner	*H. gussoneanum* 2x JB106_6
		JB113_1	Italy	Europe/SW Asia/Mediterranean	Sabine Jakob, Frank Blattner	*H. gussoneanum* 2x JB113_1
		JB157	USA	Europe/SW Asia/Mediterranean	Sabine Jakob, Frank Blattner	*H. gussoneanum* 2x JB157
	4x	H00064 (NGB06507)	Tajikistan	Europe/SW Asia/Mediterranean	Nordic Genetic Resource Centre	*H. gussoneanum* 4x H00064_A, B
		H00818 (NGB90241)	Iran	Europe/SW Asia/Mediterranean	Nordic Genetic Resource Centre	*H. gussoneanum* 4x H00818_A, B
		BCC2011 (H00821, NGB6521)	Turkey	Europe/SW Asia/Mediterranean	Barley Core Collection	*H. gussoneanum* 4x BCC2011_A, B
		BCC2013 (H00824, NGB6522)	Iran	Europe/SW Asia/Mediterranean	Barley Core Collection	*H. gussoneanum* 4x BCC2013_A, B
		01C0509096_2	Georgia	Europe/SW Asia/Mediterranean	Res. Inst. Crop Production Prague, Czech Republik	*H. gussoneanum* 4x 01C96_A, B
		H00081 (NGB06509)	Afghanistan	Europe/SW Asia/Mediterranean	Nordic Genetic Resource Centre	*H. gussoneanum* 4x H00081_A, B
Hordeum marinum Huds	2x	BCC2006 (H00546, NGB90345)	Spain	Europe/SW Asia/Mediterranean	Barley Core Collection	*H. marinum* BCC2006
		BCC2001 (H00090, NGB6821)	Greece	Europe/SW Asia/Mediterranean	Barley Core Collection	*H. marinum* BCC2001
		JB069_3	France	Europe/SW Asia/Mediterranean	Sabine Jakob, Frank Blattner	*H. marinum* JB069_3
Hordeum murinum L. subsp. *glaucum* (Steud.) Tzvelev	2x	PI218078	Pakistan	Europe/SW Asia/Mediterranean	GRIN USDA Aberdeen, USA	*H. murinum* 2x PI218078_A, B, C
		BCC2002 (H00219)	Tunisia	Europe/SW Asia/Mediterranean	Barley Core Collection	*H. murinum* 2x BCC2002_A, B, C
		PI223371 (CN064124)	Iran	Europe/SW Asia/Mediterranean	GRIN USDA Aberdeen, USA	*H. murinum* 2x PI223371_A, B
Hordeum murinum L. subsp. *murinum*	4x	JB097	Germany	Europe/SW Asia/Mediterranean	Sabine Jakob, Frank Blattner	*H. murinum* 4x JB097_A, B
		BCC2009 (H00721)	Denmark	Europe/SW Asia/Mediterranean	Barley Core Collection	*H. murinum* 4x BCC2009_A, B, C
		H00217 (NGB06870)	Germany	Europe/SW Asia/Mediterranean	Nordic Genetic Resource Centre	*H. murinum* 4x H00217_A, B, C
Hordeum murinum L. subsp. *leporinum* (Link) Arcang.	4x	BCC2007 (H00561)	Spain	Europe/SW Asia/Mediterranean	Barley Core Collection	*H. murinum* 4x BCC2007
		BCC2008 (H00591)	Greece	Europe/SW Asia/Mediterranean	Barley Core Collection	*H. murinum* 4x BCC2008
		PI244767	Iran	Europe/SW Asia/Mediterranean	GRIN USDA Aberdeen, USA	*H. murinum* 4x PI244767
		GRA1097	Bulgaria	Europe/SW Asia/Mediterranean	Gene bank IPK	*H. murinum* 4x GRA1097
	6x	F2107	Uzbekistan	Europe/SW Asia/Mediterranean	Reinhard Fritsch	*H. murinum* 6x F2107

Species	Ploidy	Accession number	Country	Species distribution	Material source	Topo6 type
		H00812 (NGB06878)	Turkey	Europe/SW Asia/Mediterranean	Nordic Genetic Resource Centre	*H. murinum* 6x H00812_A, B, C
		PI211046	Afghanistan	Europe/SW Asia/Mediterranean	GRIN USDA Aberdeen, USA	*H. murinum* 6x PI211046_A, B
Hordeum muticum J. Presl	2x	BCC2014 (H00958)	Bolivia	South America	Barley Core Collection	*H. muticum* BCC2014
		H01837 (NGB07303)	Argentina	South America	Nordic Genetic Resource Centre	
		H06457a (NGB90357)	Argentina	South America	Nordic Genetic Resource Centre	
		H06459 (NGB90358)	Argentina	South America	Nordic Genetic Resource Centre	
		H06468 (NGB90359)	Argentina	South America	Nordic Genetic Resource Centre	
		H06470 (NGB90360)	Argentina	South America	Nordic Genetic Resource Centre	
		BCC2042 (H01784, GRA982)	Argentina	South America	Barley Core Collection	*H. muticum* BCC2042
		H06446 (NGB90060)	Argentina	South America	Nordic Genetic Resource Centre	
		H00955 (NGB15689)	Bolivia	South America	Nordic Genetic Resource Centre	*H. muticum* H00955
		H00957 (NGB08566)	Bolivia	South America	Nordic Genetic Resource Centre	*H. muticum* H00957
Hordeum parodii Covas	6x	BCC2025 (H01146)	Argentina	South America	Barley Core Collection	*H. parodii* BCC2025_A, B, C
		BCC2066 (H06328)	Argentina	South America	Barley Core Collection	*H. parodii* BCC2066_A, B, C
		JB004_1	Argentina	South America	Sabine Jakob, Frank Blattner	*H. parodii* JB004_1_A, B
		JB019A_2	Argentina	South America	Sabine Jakob, Frank Blattner	*H. parodii* JB019A2_A, B, C
Hordeum patagonicum (Haumann) Covas	2x	JB044Bd	Argentina	South America	Sabine Jakob, Frank Blattner	*H. patagonicum* JB044Bd
		JB319A_1	Argentina	South America	Sabine Jakob, Frank Blattner	
		JB024_1	Argentina	South America	Sabine Jakob, Frank Blattner	*H. patagonicum* JB024_1
		JB306C_1	Argentina	South America	Sabine Jakob, Frank Blattner	
		BCC2064 (H06051)	Argentina	South America	Barley Core Collection	*H. patagonicum* BCC2064
		JB287C_3	Argentina	South America	Sabine Jakob, Frank Blattner	
		JB303B_1	Argentina	South America	Sabine Jakob, Frank Blattner	*H. patagonicum* JB303B_1
		JB303B_4	Argentina	South America	Sabine Jakob, Frank Blattner	
		H01248	Argentina	South America	Nordic Genetic Resource Centre	*H. patagonicum* H01248
		H01535	Argentina	South America	Nordic Genetic Resource Centre	*H. patagonicum* H01535
		JB015C_1	Argentina	South America	Sabine Jakob, Frank Blattner	*H. patagonicum* JB015C_1
		JB318A_4	Argentina	South America	Sabine Jakob, Frank Blattner	*H. patagonicum* JB138A_4
		JB052Ba	Argentina	South America	Sabine Jakob, Frank Blattner	*H. patagonicum* JB052Ba
		BCC2033 (H01358)	Argentina	South America	Barley Core Collection	*H. patagonicum* BCC2033
Hordeum procerum Nevski	6x	BCC2024 (H01136)	Argentina	South America	Barley Core Collection	*H. procerum* BCC2024_A, B, C
		BCC2040 (H01781)	Argentina	South America	Barley Core Collection	*H. procerum* BCC2040_A, B, C
		JB221A_2	Argentina	South America	Sabine Jakob, Frank Blattner	*H. procerum* JB221A2_A, B, C
		JB223A_3	Argentina	South America	Sabine Jakob, Frank Blattner	*H. procerum* JB223A3_A, B, C
Hordeum pubiflorum Hook.	2x	H01238 (NGB08538)	Argentina	South America	Nordic Genetic Resource Centre	*H. publiflorum* H01238
		JB027B_5	Argentina	South America	Sabine Jakob, Frank Blattner	
		JB059B_2a	Argentina	South America	Sabine Jakob, Frank Blattner	
		JB460B_3	Argentina	South America	Sabine Jakob, Frank Blattner	
		JB463B_2	Argentina	South America	Sabine Jakob, Frank Blattner	
		JB463B_4	Argentina	South America	Sabine Jakob, Frank Blattner	
		JB020_2	Argentina	South America	Sabine Jakob, Frank Blattner	*H. publiflorum* JB020_2
		JB033Ba	Argentina	South America	Sabine Jakob, Frank Blattner	
		JB323_4	Argentina	South America	Sabine Jakob, Frank Blattner	
		JB447A_1	Argentina	South America	Sabine Jakob, Frank Blattner	
		JB035C_1	Argentina	South America	Sabine Jakob, Frank Blattner	*H. publiflorum* JB035C_1
		JB312A_1	Argentina	South America	Sabine Jakob, Frank Blattner	
		JB053Ba	Argentina	South America	Sabine Jakob, Frank Blattner	*H. publiflorum* JB035Ba
		JB053Bd	Argentina	South America	Sabine Jakob, Frank Blattner	
		BCC2068 (H06687)	Bolivia	South America	Barley Core Collection	*H. publiflorum* BCC2068
		JB021_5	Argentina	South America	Sabine Jakob, Frank Blattner	*H. publiflorum* JB021_5
		JB291_2	Argentina	South America	Sabine Jakob, Frank Blattner	*H. publiflorum* JB291_2
Hordeum pusillum Nutt.	2x	CN27877	USA	North America	AGR, Saskatoon, Canada	*H. pusillum* CN27877
		Clho15663	USA	North America	GRIN USDA Aberdeen, USA	
		CN27888	USA	North America	AGR, Saskatoon, Canada	
		CN28655	USA	North America	AGR, Saskatoon, Canada	
		CN32799	USA	North America	AGR, Saskatoon, Canada	

Species	Ploidy	Accession number	Country	Species distribution	Material source	Topo6 type
		CN32803	USA	North America	AGR, Saskatoon, Canada	
		CN028654	USA	North America	AGR, Saskatoon, Canada	
		BCC2043 (H01906, GRA1176)	USA	North America	Barley Core Collection	*H. pusillum* BCC2043
		CN27810	USA	North America	AGR, Saskatoon, Canada	
		CN27814	USA	North America	AGR, Saskatoon, Canada	
		CN27885	USA	North America	AGR, Saskatoon, Canada	
		CN27886	USA	North America	AGR, Saskatoon, Canada	
		BCC2049 (H02038)	USA	North America	Barley Core Collection	*H. pusillum* BCC2049
Hordeum roshevitzii Bowden	2x	BCC2069 (H07202)	China	Central Asia	Barley Core Collection	*H. roshevitzii* BCC2069
		H07039 (NGB06891)	China	Central Asia	Nordic Genetic Resource Centre	
		H07046 (NGB06892)	China	Central Asia	Nordic Genetic Resource Centre	
		H07437 (NGB08587)	China	Central Asia	Nordic Genetic Resource Centre	
		H07754 (NGB90149)	China	Central Asia	Nordic Genetic Resource Centre	
		H08787 (NGB90068)	China	Central Asia	Nordic Genetic Resource Centre	
		H09152 (NGB90388)	China	Central Asia	Nordic Genetic Resource Centre	
		H09154 (NGB90389)	China	Central Asia	Nordic Genetic Resource Centre	
		H09157 (NGB90390)	China	Central Asia	Nordic Genetic Resource Centre	
		M4341	Mongolia	Central Asia	Karsten Wesche	
		M4352	Mongolia	Central Asia	Karsten Wesche	
		H07421a (NGB90386)	China	Central Asia	Nordic Genetic Resource Centre	*H. roshevitzii* H07421a
		H07879 (NGB90387)	China	Central Asia	Nordic Genetic Resource Centre	
		H10070 (NGB90391)	Russia	Central Asia	Nordic Genetic Resource Centre	
		H07883 (NGB90150)	China	Central Asia	Nordic Genetic Resource Centre	
		H09194 (NGB90621)	China	Central Asia	Nordic Genetic Resource Centre	*H. roshevitzii* H09194
		BCC2015 (H10070)	Russia	Central Asia	Barley Core Collection	*H. roshevitzii* BCC2015
Hordeum secalinum Schreb.	4x	BCC2004 (H00296)	Spain	Europe/SW Asia/Mediterranean	Barley Core Collection	*H. secalinum* BCC2004_A
		GRA1016	France	Europe/SW Asia/Mediterranean	Gene bank IPK	*H. secalinum* GRA1016_A, B
		JB104_1	Germany	Europe/SW Asia/Mediterranean	Sabine Jakob, Frank Blattner	*H. secalinum* JB104_1_A, B
		JB105_4	Germany	Europe/SW Asia/Mediterranean	Sabine Jakob, Frank Blattner	*H. secalinum* JB105_4_A, B
Hordeum stenostachys Godr.	2x	H01780 (NGB90151)	Argentina	South America	Nordic Genetic Resource Centre	*H. stenostachys* H01780
		H06431 (NGB90070)	Argentina	South America	Nordic Genetic Resource Centre	
		H06484 (NGB90072)	Argentina	South America	Nordic Genetic Resource Centre	
		JB216_2	Argentina	South America	Sabine Jakob, Frank Blattner	
		JB217B_2	Argentina	South America	Sabine Jakob, Frank Blattner	
		JB218_5	Argentina	South America	Sabine Jakob, Frank Blattner	
		JB219B_2	Argentina	South America	Sabine Jakob, Frank Blattner	
		JB241_1	Argentina	South America	Sabine Jakob, Frank Blattner	
		JB254_1	Argentina	South America	Sabine Jakob, Frank Blattner	
		JB254_5	Argentina	South America	Sabine Jakob, Frank Blattner	
		JB258_1	Argentina	South America	Sabine Jakob, Frank Blattner	
		JB258_2	Argentina	South America	Sabine Jakob, Frank Blattner	
		JB267_1	Argentina	South America	Sabine Jakob, Frank Blattner	
		BCC2021 (H01108)	Argentina	South America	Barley Core Collection	*H. stenostachys* BCC2021
		CN27345	Argentina	South America	AGR, Saskatoon, Canada	
		JB199A_1	Argentina	South America	Sabine Jakob, Frank Blattner	
		JB211_2	Argentina	South America	Sabine Jakob, Frank Blattner	
		JB213_5	Argentina	South America	Sabine Jakob, Frank Blattner	
		JB214_4	Argentina	South America	Sabine Jakob, Frank Blattner	*H. stenostachys* JB214_4
Hordeum tetraploidum Covas	4x	JB048C_2b	Argentina	South America	Sabine Jakob, Frank Blattner	*H. tetraploidum* JB048C_2b_A, B
		JB010_4	Argentina	South America	Sabine Jakob, Frank Blattner	*H. tetraploidum* JB010_4_A, B, C
		JB026a	Argentina	South America	Sabine Jakob, Frank Blattner	*H. tetraploidum* JB026a_A, B
		JB029B_4	Argentina	South America	Sabine Jakob, Frank Blattner	*H. tetraploidum* JB029B_4_A, B
Hordeum vulgare L. subsp. *spontaneum*	2x	F2097	Uzbekistan	Europe/SW Asia/Mediterranean	Reinhard Fritsch	*H. vulgare* subsp. *spontaneum* F2097
		F2131	Uzbekistan	Europe/SW Asia/Mediterranean	Reinhard Fritsch	*H. vulgare* subsp. *spontaneum* F2131_A, B
Dasypyrum villosum (L.) P. Candargy	2x	GRA1020	Italy	Europe/SW Asia/Mediterranean	Gene bank IPK	*Dasypyrum villosum* GRA1020
Eremopyrum triticeum (Gaertn.) Nevski	2x	GRA2250	Kazakhastan	Central Asia	Gene bank IPK	*Eremopyrum triticeum* GRA2250

Species	Ploidy	Accession number	Country	Species distribution	Material source	Topo6 type
Psathyrostachys juncea (Fisch.) Nevski	2x	GRA 692	Mongolia	Central Asia	Gene bank IPK	*Psathyrostachys juncea* GRA0692
Secale strictum (C.Presl) C.Presl subsp. *kuprijanovii* (Grossh.) K.Hammer	2x	R 1108	Kazakhastan	Central Asia	Gene bank IPK	*Secale strictum* R1108
Secale vavilovii Grossh.	2x	R1027	Italy	Europe/SW Asia/Mediterranean	Gene bank IPK	*Secale vavilovii* R1027
Taeniatherum caput-medusae (L.) Nevski	2x	GRA1126	Tajikistan	Central Asia	Gene bank IPK	*Taeniatherum caput-medusae* GRA1126
Triticum monococcum L. var. *vulgare* Körn.	2x	TRI13061	Turkey	Europe/SW Asia/Mediterranean	Gene bank IPK	*Triticum monococcum* TRI13061
Triticum urartu Tumanian ex Gandilyan var. *spontaneoalbum* Tumanian	2x	TRI17921 (PI427516)	Turkey	Europe/SW Asia/Mediterranean	USDA	*Triticum urartu* TRI17921
